# Insight into infrageneric circumscription through complete chloroplast genome sequences of two *Trillium* species

**DOI:** 10.1093/aobpla/plw015

**Published:** 2016-03-01

**Authors:** Sang-Chul Kim, Jung Sung Kim, Joo-Hwan Kim

**Affiliations:** Department of Life Science, Gachon University, Seongnamdaero 1342, Seongnam-si, Gyeonggi-do 461-701, Korea

**Keywords:** Chloroplast genome, comparative genomics, gene duplication, single inversion, *Trillium maculatum*, *Trillium tschonoskii*, *trnI*-CAU

## Abstract

Genomic events including gene loss, duplication, pseudogenization and rearrangement in plant genomes are valuable sources for exploring and understanding the process of evolution in angiosperms. The family Melanthiaceae is distributed in temperate regions of the Northern Hemisphere and divided into five tribes (Heloniadeae, Chionographideae, Xerophylleae, Melanthieae and Parideae) based on the molecular phylogenetic analyses. At present, complete chloroplast genomes of the Melanthiaceae have been reported from three species. In the previous genomic study of Liliales, a *trnI*-CAU gene duplication event was reported from *Paris verticillata*, a member of Parideae. To clarify the significant genomic events of the tribe Parideae, we analysed the complete chloroplast genome sequences of two *Trillium* species representing two subgenera: *Trillium* and *Phyllantherum*. In *Trillium tschonoskii* (subgenus *Trillium*), the circular double-stranded cpDNA sequence of 156 852 bp consists of two inverted repeat (IR) regions of 26 501 bp each, a large single-copy (LSC) region of 83 981 bp and a small single-copy (SSC) region of 19 869 bp. The chloroplast genome sequence of *T. maculatum* (subgenus *Phyllantherum*) is 157 359 bp in length, consisting of two IRs (25 535 bp), one SSC (19 949 bp) and one LSC (86 340 bp), and is longer than that of *T. tschonoskii*. The results showed that the cpDNAs of Parideae are highly conserved across genome structure, gene order and contents. However, the chloroplast genome of *T. maculatum* contained a 3.4-kb inverted sequence between *ndhC* and *rbcL* in the LSC region, and it was a unique feature for subgenera *Phyllantherum*. In addition, we found three different types of gene duplication in the intergenic spacer between *rpl23* and *ycf2* containing *trnI*-CAU, which were in agreement with the circumscription of subgenera and sections in Parideae excluding *T. govanianum*. These genomic features provide informative molecular markers for identifying the infrageneric taxa of *Trillium* and improve our understanding of the evolution patterns of Parideae in Melanthiaceae.

## Abstract (precis)

To clarify the significant genomic events of the tribe Parideae, we analysed the complete chloroplast genome sequences of two *Trillium* species representing two subgenera: *Trillium* and *Phyllantherum*. The results showed that the cpDNAs of Parideae are highly conserved across genome structure, gene order and contents. However, the chloroplast genome of *T. maculatum* contained a 3.4kb inverted sequence between *ndhC* - *rbcL* in the LSC region. In addition, we found three different types of *trnI*-CAU duplication. These genomic features provide informative molecular markers for identifying the infrageneric taxa of *Trillium* and improve our understanding of the evolution patterns of Parideae.

## Introduction

The chloroplast that characterizes all green plants (Viridiplantae) originated from an endosymbiotic event between independent living cyanobacteria and a non-photosynthetic host (Dyall *et al*. 2004). Chloroplast genomes of flowering plants are typically circular double-stranded DNA molecules, and usually contain two inverted repeat (IR) regions (IRA and IRB) separated by a large single-copy and a small single-copy (LSC and SSC, respectively) regions (Ravi *et al*. 2008). The plastid genome is mostly stable in structure, gene content and gene order across land plant lineages (Jansen *et al*. 2005). Due to this stability, it demonstrated great utility for developing phylogenetic hypotheses across the plant tree of life (Jansen *et al*. 2007; Zhang *et al*. 2011; Li *et al*. 2013). Within seed plants, plastid genomes usually contain 101–118 unique genes with the majority of those 66–82 coding for proteins involved in photosynthesis and gene expression, 29–32 of these genes code for transfer RNAs and 4 code for the ribosomal RNA genes (Jansen and Ruhlman 2012). The advance of next-generation sequencing has facilitated rapid growth of complete chloroplast genomes due to time-saving and low-cost advantages (Shendure and Ji 2008). To date, ∼500 complete chloroplast DNA genome sequence data have been released in GenBank's Organelle Genome Resources (http://www.ncbi.nlm.nih.gov/genome).

Melanthiaceae, a member of Liliales, comprises 17 genera and ∼178 species of perennial herbs that are mostly distributed in the temperate regions of the Northern Hemisphere (Zomlefer *et al*. 2001). Species of this family are characterized by their extrorse anthers and carpels bearing three distinct styles (Rudall *et al*. 2000). The family has been divided into five tribes: Heloniadeae, Chionographideae, Xerophylleae, Melanthieae and Parideae (The Angiosperm Phylogeny Group 2009). Prior to any molecular systematic analyses, Melanthiaceae were divided into several taxonomically independent families by Takhtajan (1997) due to their unique autapomorphies. Trilliaceae, which is now recognized as tribe Parideae (Trillieae), are unique in having solitary flowers, berries, membranous nectary and large chromosomes with five chromosomes as the base number. The phylogeny of species within the Trilliaceae (now Parideae) was highly debated by many researchers using molecular and morphological data (Kato *et al*. 1995; Osaloo *et al*. 1999; Osaloo and Kawano 1999; Farmer and Schilling 2002; Farmer 2006). Tribe Parideae includes three genera: *Paris*, *Trillium* and *Pseudotrillium. Paris* has 4–15 leaves in a whorl, flowers 4-merous or more and inner perianth segments that are much narrower than outer ones, while *Trillium* has only 3 leaves in a whorl, flowers 3-merous and inner perianth segments that are a little narrower than the outer ones. *Pseudotrillium* has thick, tough, heart-shaped leaves, spotted petals and flower stalks that extend until the ripe fruit touches the ground. *Trillium* has been divided into two subgenera differing in the presence of pedicel: subgenus *Trillium* (with pedicels) and *Phyllantherum* (without pedicels) (Freeman 1969, 1975). The monophyly of subgenus *Phyllantherum* was strongly supported in many previous studies (Osaloo *et al*. 1999; Osaloo and Kawano 1999; Farmer and Schilling 2002; Farmer 2006). On the other hand, subgenus *Trillium* is rendered a paraphyletic group by the inclusion of *Phyllantherum*.

Currently, complete chloroplast genomes of the Melanthiaceae have been reported from *Paris verticillata* (KJ433485; Do *et al*. 2014), *Veratrum patulum* (KF437397; Do *et al*. 2013) and *Chionographis japonica* (KF951065; Bodin *et al*. 2013), which represent three tribes of Parideae, Melanthieae and Chionographideae, respectively. In this study, we analysed complete chloroplast genome sequences of subgenera *Trillium* and *Phyllanthrum* of *Trillium* to better understand the evolution of the chloroplast genomes in tribe Parideae and across the Melanthiaceae. We analysed the sequence variation between two subgenera and proposed novel molecular markers for phylogenetic studies by comparing the two newly generated genome sequences. In addition, we characterized the *trnI*-CAU duplication event in Parideae, detected in *P. verticillata* chloroplast genome (KJ433485), to determine the origin of the repeating unit. Consequently, these results provide additional knowledge about the patterns of the chloroplast genome evolution within tribe Parideae.

## Methods

### DNA extraction, sequencing and annotation

We collected *Trillium tschonoskii* from Ulleung Island, South Korea. The voucher specimen and plant materials were deposited at the herbarium (GCU) and Medicinal Plant Resources Bank (MPRB) of Gachon University. *Trillium maculatum* was obtained from the Abraham Baldwin Agricultural College, USA (voucher No. Susan Farmer 19990006). We used silica gel-dried leaves from each species to extract total genomic DNA using the DNeasy Plant Mini Kit (Qiagen, Seoul, South Korea).

The Hiseq 2000 system was employed to sequence chloroplast genomes of *T. tschonoskii* and *T. maculatum*. Raw data were assembled using Geneious ver. 7. 1 (Biomatters Ltd, New Zealand) with default settings. After trimming the sequences, we mapped pair-end reads to the reference sequence of *P. verticillata* (KJ433485). Aligned contigs were ordered according to the reference genome and the gaps were filled via direct sequencing of polymerase chain reaction (PCR) products with newly designed primers. In addition, the ambiguous sequences including low assembly coverage regions and the borders of the four junctions between LSC, SSC and IR regions were confirmed using the Sanger method.

Complete chloroplast genomes of both species were annotated by Geneious ver. 7. 1 (Biomatters Ltd), with manual corrections for putative start and stop codons. The exon positions of protein-coding genes and intron were determined using released Liliales chloroplast genome sequences as references. All tRNA sequences were confirmed utilizing the web-based online tool of tRNAScan-SE (Schattner *et al*. 2005) with default settings to corroborate tRNA boundaries identified by Geneious. The genome maps were generated using OGDraw (OrganellarGenomeDRAW; Lohse *et al*. 2007) followed by manual modification.

### Comparison of the chloroplast genome sequences of two subgenera

The simple sequence repeats (SSRs) were analysed using Phobos Version 3.3.12 (Kraemer *et al*. 2009), with thresholds of eight repeat units for mononucleotide SSRs, four repeat units for dinucleotide, trinucleotide SSRs and three repeat units for tetranucleotide, pentanucleotide and hexanucleotide SSRs. All the detected repeats were manually verified, and the redundant results were removed. We aligned the plastid genome sequences of two *Trillium* using MAFFT (Katoh *et al*. 2002). The identified insertion/deletion mutations (indels) from the results were confirmed by reassembling the whole reads generated by HiSeq 2000. The single nucleotide polymorphisms (SNPs) were analysed using Geneious 7.1 (Kearse *et al*. 2012), and each indel and SNP were separated based on the position excluding one of IR regions. Since we are comparing only two genomes, we quantified the sequence divergence as the ratio of aligned nucleotide sites within specifically different regions (*p*-distance). Sanger sequences and assembled genomes were calculated using mean *p*-distance in MEGA 6.0 (Tamura *et al*. 2013).

Twenty-nine species, representing the two subgenera of *Trillium* in Parideae, were selected for comparative sequencing of inversion. The PCR amplification primers were designed based on the sequence comparisons among three chloroplast genome sequences of two *Trillium* species (in this study), and *P. verticillata* (KJ433485). Presence and absence PCR amplifications were carried out using various combinations of the three primers (I1F: 5′-CCC TAG GTT TTT TTC TTC AAG-3′, I1R: 5′-TTA TGT AGC TTA TCC TTT AGA CC-3′ and I2R: 5′-AGA AGG TCT ACG GTT CGA G-3′).

### *trnI*-CAU duplication pattern in the tribe Parideae

To clarify the *trnI*-CAU duplication pattern in the tribe Parideae, we designed two primers (Primer 1: 5′-GAA GAG TTC GAC CCA ATG CT-3′, Primer 2: 5′-TTA TGA AAC TCT TTG ACC CC-3′) for amplifying the intergenic spacer (IGS) region of *rpl23*-*ycf2* based on the identical sequence among the three species (*P. verticillata*, *T. maculatum* and *T. tschonoskii*). The PCR condition for IGS region of *rpl23*-*ycf2* was at initial denaturation at 94 °C for 5 min, followed by 30 cycles of denaturation at 94 °C for 1 min, annealing at 50 °C for 1 min and extension at 72 °C for 2 min, with a final extension at 72 °C for 5 min. We obtained variously sized PCR products ranging from 500 to 1200 bp, and compared the sequences of this region from 33 species covering the infrageneric classification of the tribe. Sequence editing and assembly were performed using Sequencher (ver. 5.1). The sequence alignment was initially performed using MAFFT (Katoh *et al*. 2002) and was adjusted manually.

## Results

### Comparison of the complete chloroplast genomes of subgenera *Trillium* and *Phyllantherum*

**Figure 1.**
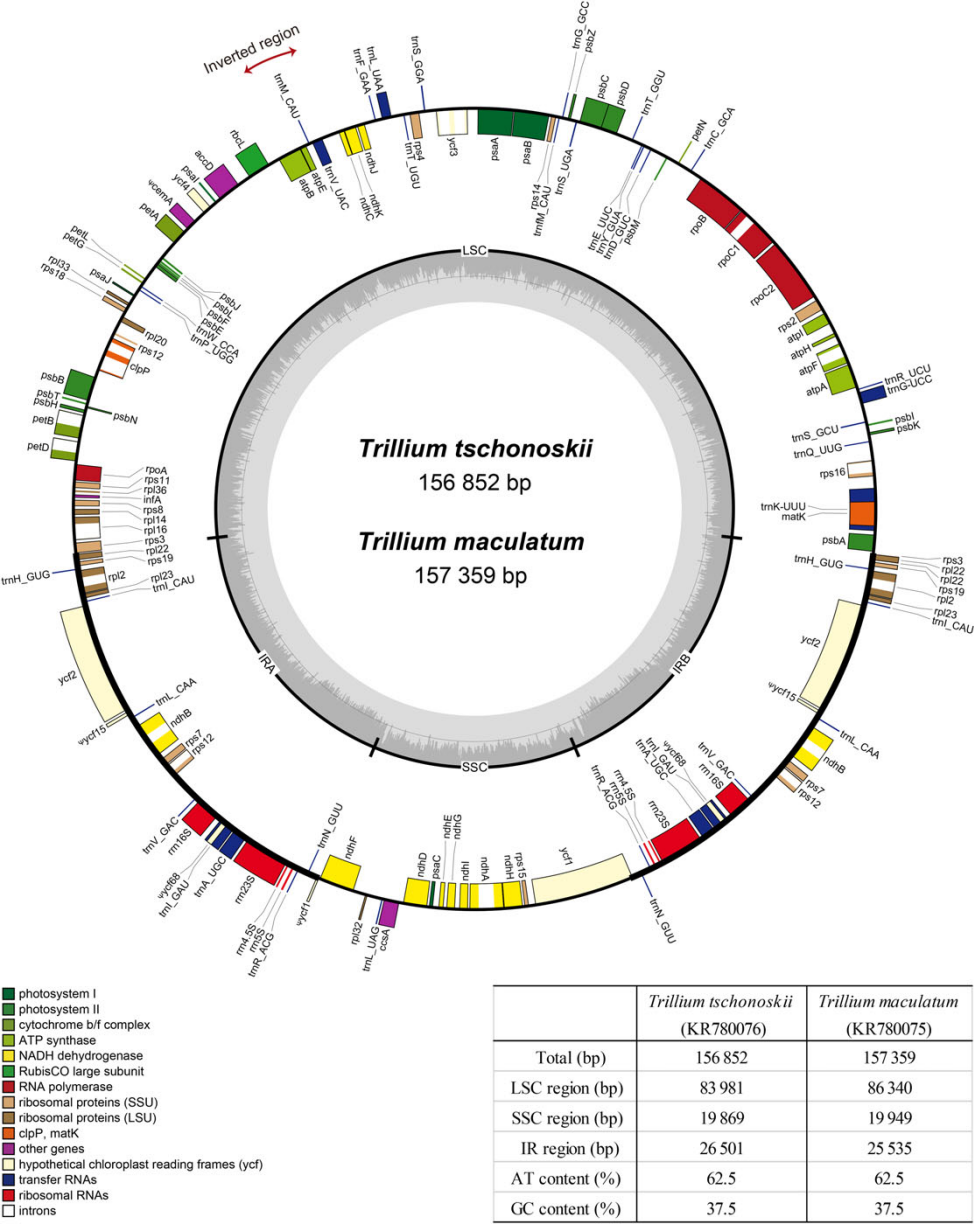
Gene maps and summary of the *T. tschonoskii* Maxim. and *T. maculatum* Raf. chloroplast genomes. IR, inverted repeat; LCS, large single-copy region; SSC, small single-copy region.

We sequenced the complete chloroplast genome sequence of two *Trillium* species, *T. tschonoskii* (subgenera *Trillium*; GenBank accession number KR780076) and *T. maculatum* (subgenera *Phyllantherum*; GenBank accession number KR780075) (Fig. 1). In total, 4 292 702 (*T. tschonoskii*) and 18 348 134 (*T. maculatum*) paired-end reads were generated. Out of those, 60 805 and 246 240 reads were identified as the chloroplast genome sequences for *T. tschonoskii* and *T. maculatum*, respectively. The chloroplast genome of *T. tschonoskii* was composed of 156 852 bp in length (AT content 62.5 %), and it comprised a LSC region (83 981 bp), a SSC region (19 869 bp) and two IR regions (26 501 bp), while *T. maculatum* was 157 359 bp in length (AT content 62.5 %, 86 340 bp of LSC, 19 949 bp of SSC and 25 535 bp of IRs).

**Figure 2.**
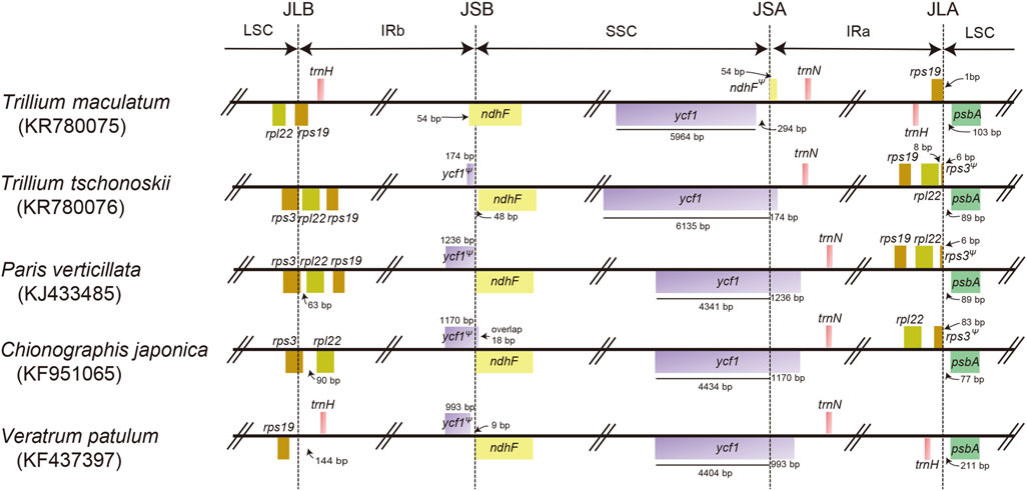
Comparison of the IR boundaries among five species within Melanthiaceae.

The gene content and order were slightly different between both species because of the *rpl22* position in the IR-LSC boundary and *trnI*-CAU duplication in IR. While the *rpl22* gene remained in the LSC region of the *T. maculatum* plastid genome, this gene was present in the IR region of *T. tschonoskii* plastid genome (Fig. 2). In total, 116 genes of *T. maculatum* were identified and consisted of 78 coding genes, 4 rRNA genes, 31 tRNA genes and 3 pseudogenes, while those of *T. tschonoskii* were 115 genes without tRNA gene duplication **[see Supporting Information—Table S1]**. In addition, *T. tschonoskii* has 7 coding genes, 4 rRNA genes, 9 tRNA genes, 2 pseudogenes, whereas *T. maculatum* has 8 coding genes, 4 rRNA genes, 8 tRNA genes, 2 pseudogenes, duplicated in the IR region, making a total of 138 genes and 137 genes presented in the *T. tschonoskii* and *T. maculatum* chloroplast genome, respectively. Among these genes, 22 intron-containing genes were found including 15 protein-coding genes and 7 tRNA genes. Among them, *ycf3* and *clpP* gene contained two introns. The *trnK*-UUU has the largest intron (*T. tschonoskii*: 2614 bp, *T. maculatum*: 2640 bp) including the *matK* gene. *Ycf15* and *ycf68* in the IR region were pseudogenized because of the presence of several internal stop codons. Furthermore, the *cemA* gene located in the LSC of both genomes was also pseudogenized.

### Characterization of single inversion in subgenus *Phyllantherum*

**Figure 3.**
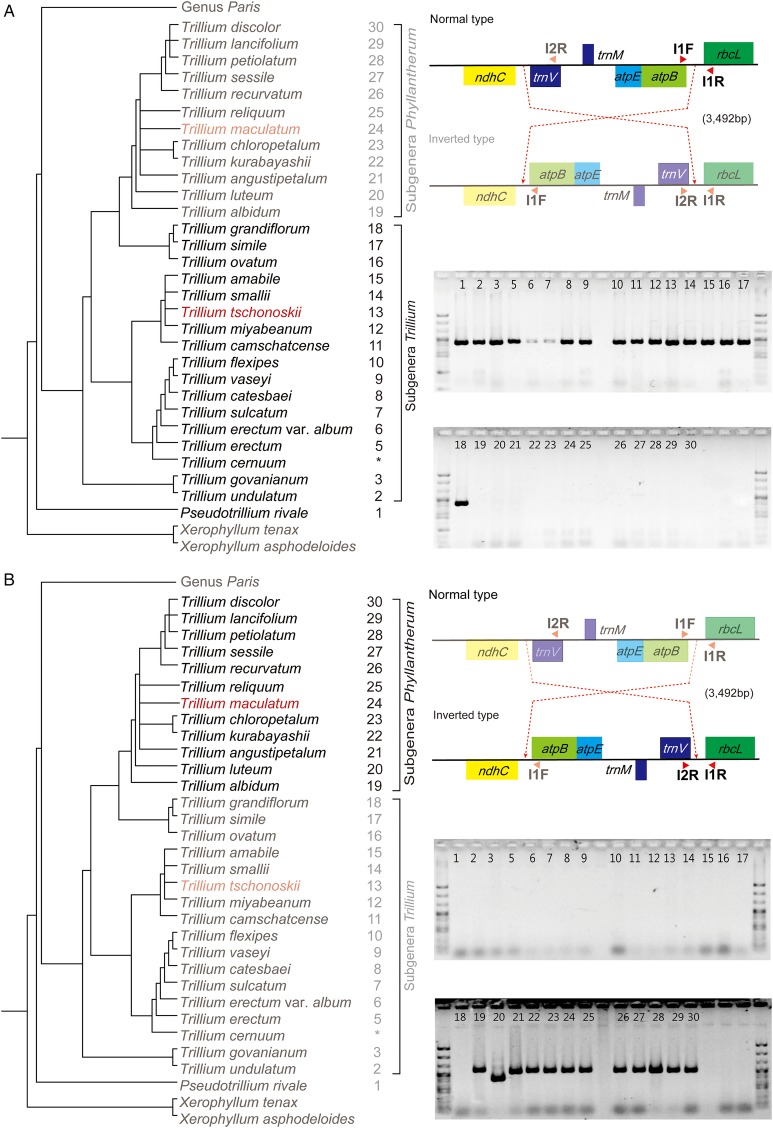
Confirmation of inversion (3492 bp) between *ndhC* and *rbcL* in the genus *Trillium*. (A) Design of primer to amplify junction regions between *atpB* and *rbcL* regions. The positions of *atpB* and *rbcL* genes in LSC regions are drawn based on the sequence assembly results of *T. tschonoskii*, *T. maculatum* in this study (red text). *The data downloaded from the NCBI. The forward primer I1F contains the sequence in *atpB* region. The sequence of the reverse primer (I1R) is located in the *rbcL* gene. Polymerase chain reaction amplification of IGS between *atpB* and *rbcL*. Relationships of Parideae lineages followed the phylogenetic trees of S. C. Kim, J. S. Kim, W. C. Mark, F. F. Michael and J. H. Kim (unpublished data). (B) Primers were designed to amplify junction regions between *trnV*-UAC and *rbcL* regions. The positions of *trnV*-UAC and *rbcL* genes in LSC regions are drawn based on the sequence assembly results of *T. tschonoskii*, *T. maculatum* in this study (red text). *The data downloaded from the NCBI. The forward primer I2R contains the sequence in *trnV*-UAC region. The sequence of the reverse primer I1R is located in the *rbcL* gene. Polymerase chain reaction amplification of IGS between *trnV*-UAC and *rbcL*. Relationships of Parideae lineages followed the phylogenetic trees of S. C. Kim, J. S. Kim, W. C. Mark, F. F. Michael and J. H. Kim (unpublished data).

Based on comparison of *T. maculatum*, *T. tschonoskii* and *P. verticillata*, a single inversion of 3.4 kb is characterized in the chloroplast genome of *T. maculatum*. This inversion is located between the *ndhC* and *rbcL* genes. We designed three different primers including I1F (5′-CCC TAG GTT TTT TTC TTC AAG-3′), I1R (5′-TTA TGT AGC TTA TCC TTT AGA CC-3′) and I2R (5′-AGA AGG TCT ACG GTT CGA G-3′) to confirm and clarify the distribution of this inversion throughout the genus *Trillium*. Specifically, the primer pairs of I1F and I1R worked only in the normal type, while I2R and I1R primer pairs were utilized for the recognized inversion type among examined species. The results showed that the inversion occurred in all examined species of the subgenera *Phyllantherum* (Fig. 3A and B).

### Indels, SNPs and SSR between two subgenera of *Trillium*

A total of 402 indels were detected between *T. maculatum* and *T. tschonoskii*, and most indels were located in the IGS regions (78.2 %). 66.2 % of the total number of indels were found in the LSC, while 22.1 and 11.7 % were present in the SSC and IR regions, respectively [Table 1, **see Supporting Information—Table S2**]. The average length of indels was 74.8 bp, and the largest indel was located in *ycf1* and *ycf2*. The frequency of 1 bp indels was 10.6 %, while 79.3 % of all indels were over 20 bp in length. In rRNA sequences, one indel of 3 bp and four indels of 5 bp were found in 16S rRNA and 23S rRNA. In addition, indel events were identified in 20 coding genes of both species (*accD*, *atpB*, *ccsA*, *cemA*, *clpP*, *infA*, *matK*, *ndhF*, *rpl2*, *rpl20*, *rpl22*, *rpl32*, *rpoC1*, *rpoC2*, *rps11*, *rps15*, *rps18*, *rps19*, *ycf1* and *ycf2*).

**Table 1.** The number and total length of insertion–deletion mutations between the chloroplast genomes of *T. tschonoskii* and *T. maculatum* in Parideae.

Region	Number of indels	Total length of indels
IGS	262	5139
Intron	51	528
Coding gene	86	3005

A total of 2861 SNPs were detected between *T. maculatum* and *T. tschonoskii* (Table 2), and 1620 SNPs were transversions. In total, 1707 (59.7 %) SNPs were located in the coding regions, and 1154 (40.3 %) were within IGS regions or within introns.

**Table 2.** Single nucleotide polymorphisms found between the plastid genomes of *T. tschonoskii* and *T. maculatum*. (A) Single nucleotide polymorphisms in coding gene. (B) Single nucleotide polymorphisms in intron. (C) Single nucleotide polymorphisms in IGS regions. Bold values represent *p*-distance >0.08.

Gene	Aligned length (bp)	No. SNP	*p*-Distance	Gene	Aligned length (bp)	No. SNP	*p*-Distance	Gene	Aligned length (bp)	No. SNP	*p*-Distance
(A)											
*psbD*	1062	2	0.002	*ycf4*	555	4	0.007	*rpl2*	828	14	0.017
*psaB*	2205	5	0.002	*cemA*	695	5	0.007	*rrn5S*	121	2	0.017
*psaA*	2253	4	0.002	*rrn23S*	2814	19	0.007	*rpl36*	114	2	0.018
*atpE*	405	1	0.002	*ndhD*	1503	10	0.007	*rrn4.5S*	103	2	0.019
*psbB*	1527	3	0.002	*ndhE*	306	2	0.007	*rrn16S*	1494	30	0.02
*petB*	648	1	0.002	*ndhI*	543	4	0.007	*rpl14*	369	9	0.024
*psbA*	1062	3	0.003	*rps15*	276	3	0.007	*ycf2*	7209	146	0.024
*ndhK*	768	2	0.003	*rpoB*	3213	25	0.008	*rps7*	468	11	0.024
*ndhC*	363	1	0.003	*psbF*	120	1	0.008	*rpl20*	387	9	0.025
*rbcL*	1434	5	0.003	*rps8*	399	3	0.008	*rps18*	363	8	0.026
*petA*	963	3	0.003	*psbN*	132	1	0.008	*rps3*	657	18	0.027
*ndhB*	1533	5	0.003	*ycf15*	234	2	0.009	*trnT*-UGU	73	2	0.027
*ccsA*	969	3	0.003	*pebT*	108	1	0.009	*trnE*-UUC	73	2	0.027
*rps16*	252	1	0.004	*rps14*	303	3	0.01	*trnC*-GCA	71	2	0.028
*atpA*	1524	6	0.004	*rps4*	606	6	0.01	*trnQ*-UUG	72	2	0.028
*atpH*	246	1	0.004	*rpoA*	1023	10	0.01	*rps12*	372	11	0.03
*psbC*	1422	6	0.004	*infA*	243	2	0.01	*rpl33*	201	7	0.035
*psbE*	252	1	0.004	*rpoC1*	2097	22	0.011	*rpl22*	387	13	0.035
*petD*	483	2	0.004	*trnS*-GCU	88	1	0.011	*rpl32*	156	6	0.04
*atpB*	1521	7	0.005	*rpl23*	282	3	0.011	*trnP*-UGG	74	3	0.041
*ycf68*	376	2	0.005	*ndhF*	2232	26	0.012	*trnI-*CAU	74	3	0.041
*ndhG*	531	3	0.006	*matK*	1554	22	0.014	*rps11*	405	19	0.048
*ndhA*	1083	7	0.006	*trnH*-GUG	74	1	0.014	***rps19***	351	24	0.084
*ndhH*	1182	7	0.006	*trnW*-CCA	74	1	0.014	***clpP***	639	65	0.111
*atpF*	555	4	0.007	*rpl16*	411	6	0.015	***ycf1***	6778	664	0.121
*rpoC2*	4140	27	0.007	*rps2*	711	12	0.017	***accD***	1566	323	0.23

Gene	Aligned length (bp)	No. SNP	*p*-Distance	Gene	Aligned length (bp)	No. SNP	*p*-Distance				
(B)											
*atpF* intron	812	14	0.018	*rpoC1* intron	714	9	0.013				
*clpP* intron 1	709	12	0.034	*rps16* intron	783	11	0.015				
*clpP* intron 2	983	29	0.012	*trnI*-GAU intron	936	2	0.002				
*ndhA* intron	1077	6	0.006	*trnK*-UUU intron	1109	34	0.012				
*ndhB* intron	695	1	0.001	*trnL*-UAA intron	538	2	0.004				
*petB* intron	823	4	0.005	*trnV*-UAC	595	4	0.007				
*petD* intron	747	5	0.007	*ycf3* intron1	737	7	0.006				
*rpl16* intron	1075	26	0.026	*ycf3* intron2	738	8	0.01				
*rpl2* intron	664	3	0.005								
IGS	Aligned length (bp)	No. SNP	*p*-Distance	IGS	Aligned length (bp)	No. SNP	*p*-Distance				
(C)											
*trnL-*CAA*_ndhB*	578	1	0.002	*rrn23S_rrn4.5S*	102	2	0.02				
*ndhB_rps7*	323	1	0.003	*rpl32_trnL-*UAG	938	16	0.02				
*atpI_rps2*	242	1	0.004	*ndhH_rps15*	110	2	0.02				
*rps12_trnV-GAC*	1905	8	0.004	*rpoB_trnC-*GCA	859	16	0.022				
*psaI_ycf4*	376	2	0.005	*rps19_trnH-*GUG	147	3	0.022				
*petB_petD*	205	1	0.005	*atpA_atpF*	92	2	0.023				
*psbE_petL*	952	6	0.006	*trnG-*GCC*_trnfM-*CAU	132	3	0.023				
*psbB_psbT*	168	1	0.006	*petA_psbJ*	1139	26	0.023				
*ycf4_cemA*	785	5	0.007	*petG_trnW-*CCA	138	3	0.023				
*rrn16S_trnI-GAU*	296	2	0.007	*rps8_rpl14*	179	4	0.023				
*trnA-*UGC*_rrn23S*	144	1	0.007	*ndhE_ndhG*	301	4	0.023				
*trnR-*ACG*_trnN-*GUU	572	4	0.007	*rps15_ycf1*	429	9	0.023				
*rps14_psaB*	132	1	0.008	*trnK-UUU_rps16*	*783*	23	0.024				
*psbN_psbH*	124	1	0.008	*rpl20_clpP*	1208	25	0.024				
*infA_rps8*	296	2	0.008	*trnL-*UAG*_ccsA*	82	2	0.025				
*trnC-*GCA*_petN*	831	8	0.01	*ndhD_psaC*	119	3	0.025				
*trnD-*GUC*_trnY-*GUA	412	4	0.01	*cemA_petA*	242	6	0.026				
*trnS-*GGA*_rps4*	307	3	0.01	*trnQ-*UUG*_psbK*	360	9	0.027				
*ycf15_trnL-*CAA	674	2	0.01	*trnG-*UCC*_trnR-*UCU	150	4	0.027				
*psbM_trnD-*GUC	1031	11	0.011	*trnT-UGU_trnL-UAA*	739	21	0.029				
*trnT-GGU_psbD*	1016	12	0.011	*rpoA_rps11*	68	2	0.029				
*ndhJ_ndhK*	89	1	0.011	*atpH_atpI*	655	19	0.031				
*psaJ_rpl33*	482	3	0.011	*rps16_trnQ-*UUG	1204	37	0.037				
*petD_rpoA*	179	2	0.011	*trnW-*CCA*_trnP-*UGG	167	6	0.037				
*ndhG_ndhI*	283	3	0.011	*ndhF_rpl32*	778	28	0.038				
*trnF-*GAA*_ndhJ*	686	8	0.012	*psbK_psbI*	396	15	0.039				
*rps2_rpoC2*	246	3	0.013	*psaC_ndhE*	380	14	0.04				
*ycf3_trnS-*GGA	759	8	0.013	*psbH_petB*	134	5	0.042				
*rps4_trnT-*UGU	320	4	0.013	*trnE-UUC_trnT-GGU*	724	26	0.044				
*trnL-UAA_trnF-GAA*	386	5	0.013	*rpl33_rps18*	200	8	0.049				
*atpF_atpH*	474	7	0.015	*clpP_psbB*	507	23	0.049				
*psbZ_trnG-*GCC	296	4	0.015	*rps11_rpl36*	151	7	0.051				
*trnM_*CAU- *atpE*	206	3	0.015	*trnS-*GCU*_trnG-*UCC	1178	57	0.052				
*psaA_ycf3*	642	10	0.016	*psbI_trnS-*GCU	124	6	0.054				
*petL_petG*	183	3	0.016	*psbC__trnS-UGA*	140	8	0.057				
*rpl36_infA*	154	2	0.016	*rpl23_trnI-*CAU	210	11	0.065				
*rpl14_rpl16*	126	2	0.016	*rpoC1_rpoB*	37	2	0.077				
*psbA_trnK-UUU*	243	10	0.017	***trnN-*GUU*_ndhF***	782	31	0.086				
*petN_psbM*	712	12	0.017	***rpl22_rps19***	115	4	0.091				
*trnY-*GUA*_trnE-*UUC	59	1	0.017	***rps3_rpl22***	80	5	0.098				
*ccsA_ndhD*	242	4	0.018	***accD_psaI***	285	20	0.099				
*rpoC2_rpoC1*	154	3	0.019	***psbT_psbN***	65	10	0.154				
*trnP-*UGG*_psaJ*	388	7	0.019	***trnH-*GUG*_rpl2***	44	8	0.186				
*rpl16_rps3*	166	3	0.019	***trnI-*CAU*_ycf2***	210	20	0.238				
*rps7_rps12*	54	1	0.019	***rbcL_accD***	2090	236	0.291				

**Figure 4.**
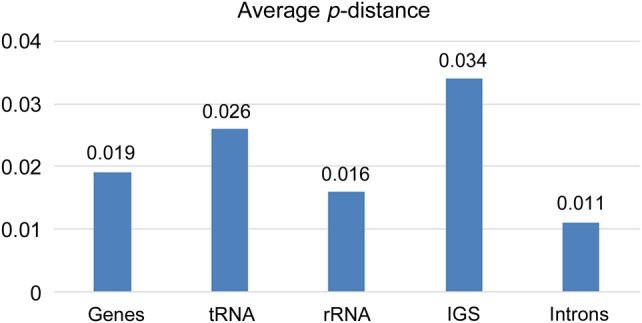
Average *p*-distance across five classes of genomic regions between two *Trillium*.

In our result of SNPs, *p*-distance values in coding regions range from 0.002 to 0.23 and the average value was 0.02. On the other hand, the average *p*-distance value in non-coding regions was 0.034. Figure 4 shows the average *p*-distance for five classes of genomic regions: protein-coding genes, tRNAs, rRNAs, IGSs and introns. The IGS divergence is almost double that of the next highest class (genes). Introns hold the lowest sequence divergence, at an average of 0.011%.

We detected SSRs longer than 8 bp in *T. maculatum*, *T. tschonoskii* and *P. verticillata* chloroplast genomes by the method of Qian *et al*. (2013). According to Qian *et al*., the threshold was set because 8 bp or longer SSRs are prone to slipstrand mispairing, which is thought to be the primary mutational mechanism causing their high level of polymorphism. In this analysis, the total number of SSRs was 204 in *P. verticillata*, 205 in *T. maculatulatum* and 213 in *T. tschonoskii* (Table 3). The most abundant type of SSR in Parideae was a mononucleotide, with 138 in *P. verticillata*, 121 in *T. maculatulatum* and 133 in *T. tschonoskii*. In addition to mononucleotide SSRs, there are 52 dinucleotide SSRs in *P. verticillata*, 57 in *T. maculatulatum* and 53 in *T. tschonoskii*. Trinucleotide SSRs were less frequent with 6, 14 and 7 in *P. verticillata*, *T. maculatum* and *T. tschonoskii*, respectively. The hexanucleotide SSRs were found only in *Trillium* species. The majority of mononucleotide repeats were A-T rich (Table 3).

**Table 3.** Number of SSRs present in the three Parideae chloroplast genomes.

Taxon	*Paris verticillata*	*Trillium maculatum*	*Trillium tschonoskii*
Genome size	157 379	157 359	156 852
No. of SSRs			
A/T	133	117	127
C/G	5	4	6
AC/GT	3	3	3
AG/CT	17	19	18
AT/TA	32	35	32
AAG/CTT		2	2
AAT/ATT	6	10	3
ACT/AGT		2	1
ATC/GAT			1
AAAG/CTTT	–	–	1
AAAT/ATTT	3	4	4
AAGG/CCTT	1	1	1
AATC/GATT	1	1	1
AATG/CATT	1	1	3
AGAT/ATCT	1	1	1
ACTAT/ATAGT	1	–	1
AAAAT/ATTTT	–	1	1
AATAT/ATATT	–	2	1
AATATG/CATATT	–	1	–
AAAATC/GATTTT	–	1	–
ATATCC/GGATAT	–	–	1
AAAAAT/ATTTTT	–	–	2
AAGACT/AGTCTT	–	–	1
AACTAC/GTAGTT	–	–	1
AAAGAG/CTCTTT	–	–	1
Total	204	205	213

### Type of *trnI*-CAU of Parideae

**Figure 5.**
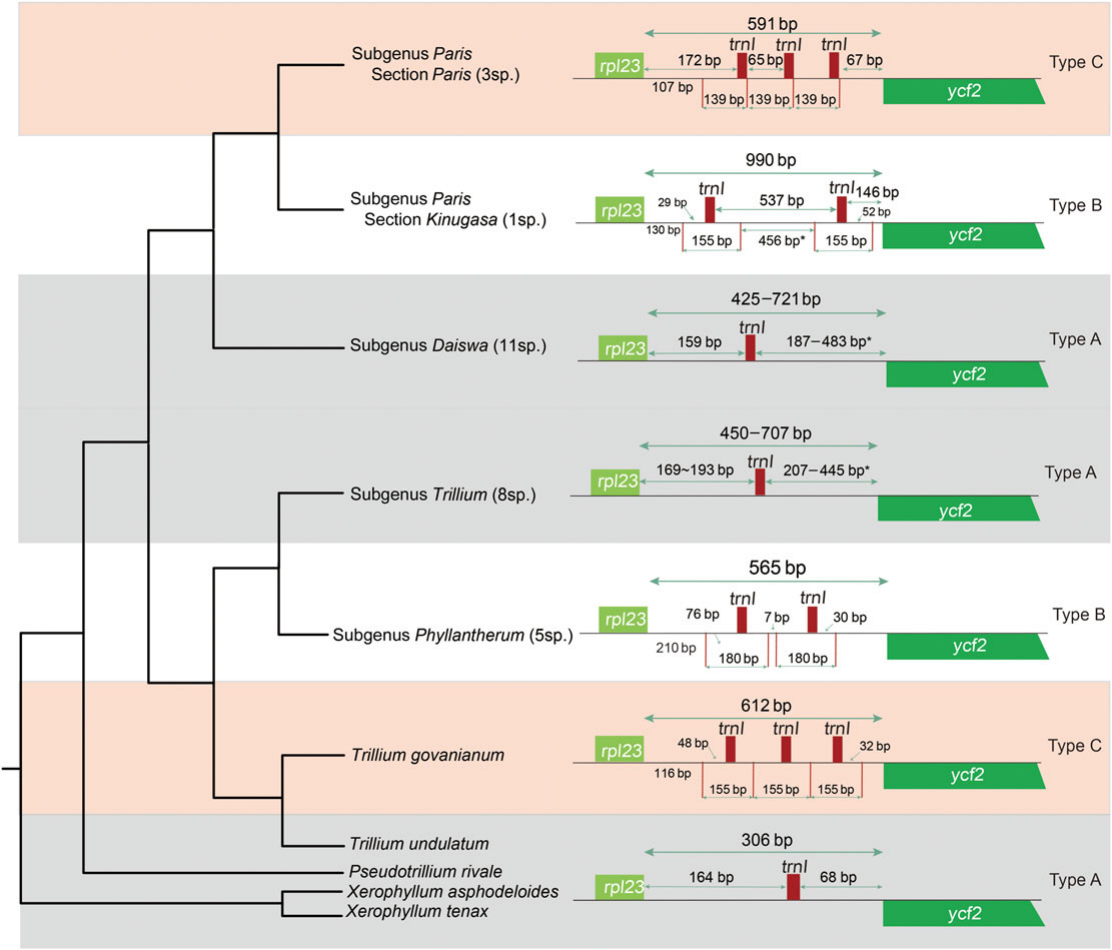
Summary of three types of *trnI*-CAU gene duplication in the tribe Parideae. *Including tandem repeats.

We compared the sequences of the IGS region between *rpl23* and *ycf2* using 33 species including *Xerophyllum* to understand the evolutionary implication of *trnI*-CAU duplication, which was reported from the *Paris* chloroplast genome (Do *et al*. 2014). Based on the results, we found that this region is of highly variable length among the species, and we distinguished three major types based on the number of copies of *trnI*-CAU (Fig. 5). Type A was composed of a single *trnI*-CAU and was found in *Xerophyllum*, *Pseudotrillium rivale* and *T. undulatum*. It was also identified in several *Trillium* and *Paris* species, but with variable lengths: in subgenus *Trillium* species, the sequences ranged from 207 to 445 bp, in which there are two tandem repeats of ‘CAG GTA TTA TCA TAC TGA AA’ (20 bp) and ‘CAT ATT ATC ATA CTG AAA’ (18 bp). Similarly, in subgenus *Daiswa* of *Paris*, there were 24 bp random tandem repeats of TAT AAC TTA ACA GGA ATC ATC GTA. Type B contained two copies of *trnI*-CAU. This type is found in subgenus *Phyllantherum* of *Trillium* and section *Kinugasa* of subgenus *Paris*. The lengths of tandem repeat sequences were 180 bp (subgenera *Phyllantherum*) and 155 bp (section *Kinugasa* of subgenus *Paris*), which included 74 bp of *trnI*-CAU. Remarkably, section *Kinugasa* (*Paris japonica*) has the longest length of IGS between *rpl23* and *ycf2* among the tribe Parideae. Type C, possessing three copies of *trnI*-CAU genes in the sequenced region, was detected in *T. govanianum* and section *Paris* of subgenus *Paris*. They included three fully repeated units including *trnI*-CAU, and the lengths were 155 and 139 bp, respectively.

## Discussion

### Comparison of complete plastid genomes of subgenera *Trillium* and *Phyllantherum*

The plastid genome structure of the two *Trillium* species, *T. maculatum* and *T. tschonoskii*, have a typical form found in most angiosperms (Zhang *et al*. 2011; Kim and Kim 2013; Li *et al*. 2013; Qian *et al*. 2013). The *T. tschonoskii* chloroplast genome was 507 bp shorter than *T.maculatum*, and we confirmed that the length variation among Parideae chloroplast genomes including *Paris verticillata* occurred by gene deletion and duplication as well as its IR expansion.

Although chloroplast genomes are considered highly conserved among land plants, sequence polymorphisms were often observed among closely related species. From the *T. tschonoskii* and *T. maculatum* chloroplast genome sequences, we confirmed that 402 indels and 2861 SNPs were present between the two species.

In addition, we found that SSRs (i.e. microsatellites), composed of 1–6 bp in length per unit, are distributed throughout both genomes. The SSRs have been accepted as one of the major molecular markers for genome variation between species or within populations due to their high polymorphism within the species and have been widely practiced for analysing plant population structure, diversity, differentiation and maternity analysis (Liu *et al*. 2013). Simple sequence repeats have successfully been applied to the study of Poaceae, Brassicaceae and Solanaceae (Provan *et al*. 1997, 1999; Bryan *et al*. 1999; Flannery *et al*. 2006). Simple sequence repeats detected in the present study will provide basic information for the further analysis of genetic diversity in Parideae.

Based on our results, the IR/LSC boundary and the IR/SSC boundary differed between the two subgenera of *Trillium*. Inverted repeat/large single-copy junction was expanded to a part of *rps3* in *T. tschonoskii*, whereas that of *T. maculatum* was found at *rps19*. The *ycf1* was completely located in SSC of *T. tschonoskii*, but a part of the *ycf1* gene was duplicated in IR of *T. maculatum*. Within the Parideae, the IR boundary pattern of *T. tschonoskii* was more similar to *P. verticillata* than *T. maculatum* (Fig. 2).

### Inversion events in Melanthiaceae

Inversions caused by the recombination between repeated sequences are considered to be a main mechanism for changes in gene order among plastid genomes (Jansen and Ruhlman 2012). Most of the reported inversions in plastid genomes are in the LSC region (Kim *et al*. 2005). In subtribe *Phaseolinae* of Fabaceae, there is a 78 kb inversion between *trnH/rpl14* and *rps19/rps8* in the chloroplast genome (Tangphatsornruang *et al*. 2010). Additionally, Kim *et al*. (2005) reported that the inversion occurred in the spacer between tRNA^Gly^ and tRNA^Ser^ genes of *Lactuca sativa*. Also, they defined two inversions that characterize Asteraceae. The two inversions were identical across all members of Asteraceae, suggesting that the inversion events are likely to occur simultaneously or within a short period of time following the origin of the family. In Campanulaceae, >50 large inversions occurred during diversification of the family, in which at least 20 occurred in *Cyphia*, and a minimum of 53 are now known in *Lobelia* (Knox 2014). Fabaceae are known to exhibit a number of unusual phenomena in their chloroplast genome: *Trifolium subterraneum* has undergone extensive genomic reconfiguration, including the loss of six genes and two introns and numerous gene order changes, attributable to 14–18 inversions (Cai *et al*. 2008).

Our results confirmed a single inversion in Melanthiaceae. It was remarkable that a single inversion of 3492 bp embedded four genes between *ndhC* and *rbcL* genes, which specifically occurred in the monophyletic subgenus *Phyllantherum* (Fig. 3). This event is thought to have occurred after the evolutionary divergence between subgenus *Phyllantherum* and subgenus *Trillium*. This new finding may be an effective molecular marker for classifying subgenera of the genus *Trillium*.

### Diverse patterns of *trnI*-CAU duplication in Parideae

Gene duplication is an important process in organellar genome evolution. Most duplicated genes occur within the IR regions due to the mechanisms underlying IR expansion and contraction (Xiong *et al*. 2009). Gene duplication in plastid genome has been reported in tRNA genes (Hipkins *et al*. 1995; Vijverberg and Bachmann 1999; Schmickl *et al*. 2009) and in some protein-coding genes. Most of the duplications can be detected only in rearranged chloroplast genomes, as in grasses, legumes and conifers. Hipkins *et al*. (1995) compared the number of direct repeats between partially duplicated *trnY*-GUA and the complete *trnY*-GUA gene in *Pseudotsuga.* They found that the length-variable region in *Pseudotsuga* comprised imperfect tandem direct repeats based on the *trnY* gene sequence. Schmickl *et al*. (2009) used the 5′-*trnL*-UAA_*trnF*-GAA region for phylogeographic reconstructions, gene diversity calculations and phylogenetic analyses among the genera *Arabidopsis* and *Boechera*. The Cruciferous taxa are characterized by these pseudogenes in at least four independent phylogenetic lineages. In addition, the tRNA gene as well as the coding gene could be confirmed by duplication events in *Jasminum* and *Menodora*, which have the duplicated *rbcL_psaI* region. Most chloroplast gene duplications outside of the IR involve tRNAs, as in the case of Oleaceae (Lee *et al*. 2007).

A total of 30–32 tRNA genes are present within the chloroplast genome of land plants (Tsudzuki *et al*. 1994; Vijverberg and Bachmann 1999), and they may be involved in chloroplast genome rearrangements through their secondary structure (Howe *et al*. 1988). These genes are dispersed throughout the genome, but five to eight genes are located in the IR (Maréchal-Drouard *et al*. 1993). We found that three major types of *trnI*-CAU gene duplication are located between *rpl23* and *ycf2* at the IR of tribe Parideae (Fig. 5). Traditionally, Parideae included two genera, *Paris* and *Trillium*; however, *Trillium* was separated into two genera *Trillium* and *Pesudotrillium* in recent classifications (Farmer and Schilling 2002). Using the various duplication patterns of *trnI*-CAU in the IR region, the infrageneric circumscription of Parideae member was strongly supported. The type of *trnI*-CAU that had been discovered in *Xerophyllum*, *Pesudotrillium* and *T. undulatum* with one *trnI*-CAU between *rpl23* and *ycf2* was seen to be similar to the ancestor of Parideae (Type A, Fig. 5). This type was found also in most chloroplast genomes of Liliales (Liu *et al*. 2012; Bodin *et al*. 2013; Do *et al*. 2013; Kim and Kim 2013). It was modified in subgenus *Trillium* of *Trillium* and subgenus *Daiswa* of *Paris* to be extended by the tandem repeat between *trnI*-CAU and *ycf2*. Type B was found in subgenus *Phyllantherum* of *Trillium* and section *Kinugasa* of subgenus *Paris* although section *Kinugasa* possessed the additional tandem repeat between *trnI*-CAU units. Type C, which was found in *T. govanianum* and section *Paris* of subgenus *Paris*, has three copies of the *trnI*-CAU gene. From the results, we suggested that duplicate events of *trnI*-CAU have occurred independently in the tribe Parideae of Melanthiaceae, and it provided useful information for determining the infrageneric circumscription. However, *T. govanianum*, which was classified into another genus *Trillidium* by Farmer and Schilling (2002) based on morphological characters and geographical distribution, was more similar to *Paris* than *Trillium*. Also, this result showed that the *trnI*-CAU gene duplication pattern of *T. govanianum* was more similar to *Paris* than *Trillium*. Interestingly, it was positioned at the same clade together with the North American species *T. undulatum* in the molecular phylogenetic tree (Farmer and Schilling 2002; S. C. Kim, J. S. Kim, W. C. Mark, F. F. Michael, J. H. Kim, unpublished data). Further studies are necessary to clarify the relationship between both species.

## Conclusions

We analysed the complete chloroplast genomes of two species of *T. tschonoskii* (subgenus *Trillium*) and *T. maculatum* (subgenus *Phyllantherum*) to verify the specific feature in the genome level. As a result, we found a 3.4 kb inverted sequence between *ndhC* and *rbcL* in the LSC region in the chloroplast genome of *T. maculatum*, which was unique to subgenus *Phyllantherum*. In addition, three different gene duplication patterns of *trnI*-CAU gene were found and they were the informative molecular markers for identifying the infrageneric taxa of *Trillium*.

## Sources of Funding

This work was supported by the National Research Foundation of Korea (NRF) Grant Foundation (MEST 2010-0029131) and Korea National Arboretum (KNA1-2-13,14-2).

## Contributions by the Authors

J.-H.K conceived and designed the experiments, S.-C.K. performed the experiments, S.-C.K. and J.S.K. analysed the data and S.-C.K. and J.-H.K. wrote the paper.

## Conflict of Interest Statement

None declared.

## Supporting Information

The following additional information is available in the online version of this article –

**Table S1.** Genes found in *Trillium tschonoskii* and *T. maculatum* chloroplast genomes.

**Table S2.** The detailed list of insertion–deletion mutations between the chloroplast genomes of *T. tschonoskii* and *T. maculatum* in Parideae.

**Table S3.** Sequences of *rpl23*_*ycf2* IGS among Parideae species.

Additional Information
