# Supplementary material for: Insight into infrageneric circumscription through complete chloroplast genome sequences of two Trillium species
Source: AoB Plants. 2016 Mar 1;8:plw015. doi: 10.1093/aobpla/plw015 (PMC4823371; doi:10.1093/aobpla/plw015)
Supplement: Additional Information [file supp_8_plw015_index.html]

Insight into infrageneric circumscription through complete chloroplast genome sequences of two Trillium species — Additional Information 

# Insight into infrageneric circumscription through complete chloroplast genome sequences of two *Trillium* species

## Additional Information

Additional Information

- Supplementary File 1 - docx file
- Supplementary File 2 - docx file
- Supplementary File 3 - doc file
