# Supplementary material for: Insight into infrageneric circumscription through complete chloroplast genome sequences of two Trillium species
Source: AoB Plants. 2016 Mar 1;8:plw015. doi: 10.1093/aobpla/plw015 (PMC4823371; doi:10.1093/aobpla/plw015)
Supplement: Additional Information [file supp_plw015_plw015supp_file1.docx]

Supporting file 1. List of genes found in *Trillium tschonoskii* and *T. maculatum* chloroplast genomes.

| Gene types | Gene products | | *T. tschonoskii* | *T. maculatum* |
| --- | --- | --- | --- | --- |
| Ribosomal RNAs | *rrn4.5*(x2)*, rrn5*(x2)*, rrn16*(x2)*, rrn23*(x2) | | 8 | 8 |
| Transfer RNAs | *trnA-*UGC (x2) ^b^*, trnC-*GCA*, trnD-*GUC*, trnE-*UUC*, trnF-*GAA*, trnfM-*CAU*, trnG-*GCC*, trnG-*UCC^b^*, trnH-*GUG(x2)*,* ***trnI-*CAU(4x/2x)***, trnI-*GAU (x2) ^b^*, trnK-*UUU^b^*, trnL-*CAA(x2)*,trnL-*UAA^b^, *trnL-*UAG*, trnM-*CAU*, trnN-*GUU(x2)*, trnP-*UGG*, trnQ-*UUG*, trnR-*ACG(x2)*, trnR-*UCU*, trnS-*GCU*, trnS-*GGA*, trnS-*UGA*, trnT-*GGU*, trnT-*UGU*, trnV-*GAC(x2)*, trnV-*UAC^b^*, trnW-*CCA*, trnY-*GUA | | 38 | 40 |
| Photosystem I | *psaA, psaB, psaC, psaI, psaJ* | | 5 | 5 |
| Photosystem II | *psbA, psbB, psbC, psbD, psbE, psbF, psbH, psbI, psbJ, psbK, psbL, psbM, psbN, psbT, psbZ* | | 15 | 15 |
| Cytochrome | *petA, petB*^b^*, petD*^b^*, petG, petL, petN* | | 6 | 6 |
| ATP synthase | *atpA, atpB, atpE, atpF*^b^*, atpH, atpI* | | 6 | 6 |
| Rubisco | *rbcL* | | 1 | 1 |
| NADH dehydrogenease | *ndhA*^b^*, ndhB* (x2) ^b^*, ndhC, ndhD, ndhE, ndhF, ndhG, ndhH, ndhI, ndhJ, ndhK* | | 12 | 12 |
| ATP-dependent protease subunit P | *clpP*^a^ | | 1 | 1 |
| Chloroplast envelope membrane protein | Ψ*cemA* | | 1 | 1 |
| Large subunit ribosomal proteins | *rpl2*(x2)^b^*, rpl14, rpl16*^b^*, rpl20, rpl22*(2x/1)*, rpl23*(x2)*, rpl32, rpl33, rpl36* | | 12 | 11 |
| Small subunit ribosomal proteins | *rps2, rps3, rps4, rps7*(x2)*, rps8, rps11, rps12* (x2) ^a^*, rps14, rps15, rps16* ^a^*, rps18, rps19*(x2) | | 15 | 15 |
| RNA polymerase | *rpoA, rpoB, rpoC1*^b^*, rpoC2* | | 4 | 4 |
| Initiation factor | *infA* | | 1 | 1 |
| Subunit of acetyl-CoA-carboxylase | *accD* | | 1 | 1 |
| C-type cytochrome synthesis gene | *ccsA* | | 1 | 1 |
| Maturase | *matK* | | 1 | 1 |
| Conserved open reading frames | *ycf1, ycf2*(x2)*, ycf3*^a^*, ycf4,* Ψ*ycf15*(x2), Ψ*ycf68*(x2) | | 9 | 9 |
| Total | |  | 137 | 138 |
| *(x2): Two gene copies in IRs.  ^a^: Gene containing two introns.  ^b^: Gene containing a single intron.  Ψ: pseudogene | |  |  |  |
