# Supplementary material for: Insight into infrageneric circumscription through complete chloroplast genome sequences of two Trillium species
Source: AoB Plants. 2016 Mar 1;8:plw015. doi: 10.1093/aobpla/plw015 (PMC4823371; doi:10.1093/aobpla/plw015)
Supplement: Additional Information [file supp_plw015_plw015supp_file2.docx]

Supporting file 2. The detailed list of Insertion-deletion mutations between the chloroplast genomes of *T. tschonoskii* and *T. maculatum* in Parideae. A: IGS region B: coding gene and intron

A.

| Region | Number of Indels | Total length of indels | Region | Number of Indels | Total length of indels |
| --- | --- | --- | --- | --- | --- |
| *psbA ~ trnK*-UUU | 1 | 6 | ***trnP*-UGG ~ *psaJ*** | 4 | 22 |
| *trnK*-UUU *~ matK* | 6 | 24 | ***psaJ ~ rpl33*** | 5 | 201 |
| *matK ~ trnK*-UUU | 3 | 42 | ***rpl33 ~ rps18*** | 2 | 38 |
| *trnK*-UUU *~ rps16* | 4 | 61 | ***rps18 ~ rpl20*** | 3 | 98 |
| *rps16 ~ trnQ-*UUG | 10 | 209 | ***rpl20 ~ clpP*** | 8 | 152 |
| *trnQ*-UUG *~ psbK* | 2 | 25 | ***clpP ~ psbB*** | 2 | 36 |
| *psbK ~ psbI* | 3 | 11 | ***psbB ~ psbT*** | 1 | 2 |
| *psbI ~ trnS*-GCU | 2 | 12 | ***psbH ~ petB*** | 1 | 15 |
| *trnS*-GCU *~ trnG*-UCC | 11 | 85 | ***petB ~ petD*** | 1 | 8 |
| *trnR-*UCU *~ atpA* | 1 | 6 | ***rps11 ~ rpl36*** | 3 | 14 |
| *atpA ~ atpF* | 1 | 5 | ***rpl36 ~ infA*** | 2 | 29 |
| *atpF ~ atpH* | 2 | 7 | ***infA ~ rps8*** | 1 | 164 |
| *atpH ~ atpI* | 8 | 38 | ***rps8 ~ rpl14*** | 1 | 6 |
| *atpI ~ rps2* | 1 | 5 | ***rpl14 ~ rpl16*** | 1 | 2 |
| *rps2 ~ rpoC2* | 2 | 6 | ***rpl16* ~ *rps3*** | 3 | 12 |
| *rpoC1 ~ rpoB* | 1 | 11 | ***rps3 ~ rpl22*** | 3 | 29 |
| *rpoB~trnC-*GCA | 3 | 131 | ***rpl22 ~ rps19*** | 2 | 71 |
| *trnC-*GCA *~ petN* | 2 | 2 | ***rps19 ~ trnH*-GUG** | 1 | 9 |
| *petN ~ psbM* | 3 | 10 | ***trnH*-GUG *~ rpl2*** | 1 | 1 |
| *psbM ~ trnD-*GUC | 3 | 13 | ***rpl23 ~ trnI*-CAU** | 2 | 41 |
| *trnD-*GUC *~ trnY-*GUA | 2 | 13 | ***trnI*-CAU *~ ycf2*** | 2 | 126 |
| *trnE-*UUC *~ trnT-*GGU | 4 | 128 | *trnT-*GGU *~ psbD* | 6 | 22 |
| *psbZ ~ trnG-*GCC | 2 | 24 | ***ycf15 ~ trnL*-CAA** | 1 | 471 |
| *trnG-*GCC *~ trnfM-*CAU | 1 | 1 | ***trnL*-CAA *~ ndhB*** | 1 | 7 |
| *trnfM-CAU ~ rps14* | 1 | 3 | ***rps12 ~ trnV*-GAC** | 5 | 31 |
| *psaA ~ ycf3* | 1 | 2 | ***Rrn16 ~ trnI*-GAU** | 1 | 1 |
| *ycf3 ~ trnS-GGA* | 6 | 123 | ***rrn23 ~ rrn4.5*** | 1 | 4 |
| *trnS-GGA ~ rps4* | 2 | 7 | ***rrn4.5 ~ rrn5*** | 1 | 5 |
| *trnT-*UGU *~ trnL-*UAA | 3 | 21 | ***rrn5 ~ trnR*-ACG** | 1 | 5 |
| *trnL-*UAA *~ trnF-*GAA | 2 | 8 | ***trnR*-ACG *~ trnN*-GUU** | 2 | 10 |
| *trnF-*GAA *~ ndhJ* | 7 | 28 | ***trnN*-GUU *~ ndhF*** | 3 | 423 |
| *trnV-*UAC *~ trnM-*CAU | 1 | 6 | ***ndhF ~ rpl32*** | 5 | 43 |
| *trnM-*CAU *~ atpE* | 2 | 12 | ***rpl32 ~ trnL*-UAG** | 12 | 124 |
| *rbcL ~ accD* | 29 | 1280 | ***ccsA ~ ndhD*** | 3 | 15 |
| *accD ~ psaI* | 4 | 82 | ***psaC ~ ndhE*** | 3 | 32 |
| *psaI ~ ycf4* | 1 | 6 | ***ndhE ~ ndhG*** | 5 | 126 |
| *cemA ~ petA* | 2 | 7 | ***ndhG ~ ndhI*** | 3 | 20 |
| *petA ~ psbJ* | 4 | 19 | ***ndhI ~ ndhA*** | 2 | 8 |
| *psbE ~ petL* | 3 | 18 | ***ndhH ~ rps15*** | 2 | 10 |
| *petG ~ trnW-CCA* | 2 | 7 | ***rps15 ~ ycf1*** | 3 | 48 |
| *trnW*-CCA *~ trnP*-UGG | 1 | 6 | ***ycf1 ~ trnN*-GUU** | 4 | 148 |

B.

| Region | Number of Indels | Total length of indels | Region | Number of Indels | Total length of indels |
| --- | --- | --- | --- | --- | --- |
| *matK* | 1 | 6 | ***rps19*** | 2 | 66 |
| *rpoC1* | 4 | 24 | ***ycf2*** | 19 | 1230 |
| *accD* | 12 | 126 | ***rrn16*** | 1 | 3 |
| *cemA* | 1 | 1 | ***rrn23*** | 4 | 5 |
| *clpP* | 1 | 54 | ***rpl20*** | 1 | 33 |
| *infA* | 2 | 42 | ***rps16* intron** | 2 | 30 |
| *ndhF* | 2 | 9 | ***atpF* intron** | 5 | 31 |
| *ccsA* | 1 | 3 | ***rpoC1* intron** | 3 | 8 |
| *ycf1* | 25 | 1259 | ***ycf3* intron** | 3 | 17 |
| *atpB* | 2 | 24 | ***clpP* intron** | 12 | 303 |
| *rpl2* | 1 | 6 | ***petB* intron** | 2 | 11 |
| *rpl22* | 2 | 18 | ***petD* intron** | 2 | 7 |
| *rpoC2* | 2 | 30 | ***rpl16* intron** | 12 | 83 |
| *rps11* | 1 | 6 | ***rps12* intron** | 1 | 5 |
| *rps15* | 1 | 9 | ***trnI*-GAU intron** | 1 | 1 |
| *rps18* | 1 | 51 | ***ndhA* intron** | 4 | 11 |
|  |  |  | ***trnL-*UAA intron** | 4 | 21 |
