# Supplementary material for: Insight into infrageneric circumscription through complete chloroplast genome sequences of two Trillium species
Source: AoB Plants. 2016 Mar 1;8:plw015. doi: 10.1093/aobpla/plw015 (PMC4823371; doi:10.1093/aobpla/plw015)
Supplement: Additional Information [file supp_plw015_plw015supp_file3.doc]

Supplementary Table S3. Sequences of *rpl23*_*ycf2* IGS among Parideae species.

The green, red, and bluish green shaded sequences indicate the *rpl23*, *trnI*-CAU and *ycf2*, respectively. The bold-font sequences show the repeat units, which include *trnI*-CAU. The asterisks above the sequences indicate the start nucleotide of the repeat unit.

**Type A**

>*Xerophyllum tenax*

GACATTAGAAGTATATTGATTGTTCCCCAATAACCGAATACTTTTTTCTGTAAATACTGCATATTTGATTCCATCCATAAATCCATTTTCTTCCCTATGAGTTCCAGTATCGATAAGAATTCTAGTTCTTACTGTTCATATGTTATGGTATGAATATACCATACCAATTCGTTATGTATGGATGATGAGATTCCATTGATACAGAGCCAATTCCAATAGACTTATTGAACGTTCCATTGGCGTGCATCCAGCAGGAATTGAACCTACGAATTTGCCAATTATGAGTTGGGCGCTTTAACCATTCAGCCATGGATGCTTAACAGGGATCATCGTACATCGTGAATAACCAAATTCCAATTGAAATGAAATCTTTAGGAGGAATCAATGAAACGACATCAATTCAAATCCTGGATCTTCGAATTGAGAGAGATATTGAGAGAGATCAAGAATTCTCACTATTTCTTAGATTCATGGATCAAATTCGATTCAGTGGGATCTTTCACTCACATTTT

>*Xerophyllum asphodeloides*

GACATTAGAAGTATATTGATTGTTCCCCAATAACCGAATACTTTTTTCTGTAAATACTGCATATTTGATTCCATCCATAAATCCATTTTCTTCCCTATGAGTTCCAGTATCGATAAGAATTCTAGTTCTTACTGTTCATATGTTATGGTATGAATATACCATACCAATTCGTTATGTATGGATGATGAGATTCCATTGATACAGAGCCAATTCCAATAGACTTATTGAACGTTCCATTGGCGTGCATCCAGCAGGAATTGAACCTACGAATTTGCCAATTATGAGTTGGGCGCTTTAACCATTCAGCCATGGATGCTTAACAGGGATCATCGTACATCGTGAATAACCAAATTCCAATTGAAATGAAATCTTTAGGAGGAATCAATGAAACGACATCAATTCAAATCCTGGATCTTCGAATTGAGAGAGATATTGAGAGAGATCAAGAATTCTCACTATTTCTTAGATTCATGGATCAAATTCGATTCAGTGGGATCTTTCACTCACATTTT

>*Pseudotrillium rivale*

GACATTAGAAGTATATTGATTGTTCCCCAATAACCGAATACTTTTTTCTGTAAATACTGCATATTTGATTCCATCCATAAATCCATTTTCTTCCCTATGAGTTCCAGTATCGATAAGAATTCTAGTTCTTACTGTTCATATGTTATGGTATGAATATACCATATCAATTCGTTATGTATGGATGATGAAATTCCATTGATACAGAGCCAATTCCAATAGACTTATTGAACGTTCCATTAGCGTGCATCCAGCAGGAATTGAACCTACGAATTTGCCAATTATGAGTTGGGCGCTTTAACCATTCAGCCATGGATGCTTAACAGGGATTATCGTAAATAACCAAATTCCAATTGAAATAAAATATTTAGGAGGAATCAATGAGACGACATCAATTCAAATCCTGGATCTTCGAATTGAGAGAGATATTGAGAGAGATCAAGAATTCTCACTATTTCTTAGATTCATGGATCAAATTCGATTCAGTGGGATCTTTCACTCACATTTT

>*Trillium undulatum*

GACATTAGAAGTATATTGATTGTTCCCCAATAACCGAATACTTTTTTCTGTAAATACTGCATATTTGATTCCATCCATAAATCCATTTTCTTCCCTATGAGTTCCAGTATCGATAAGAATTCTAGTTCTTACTGTTCATATGTTATGGTATGAATATACCATATCAATTCGTTATGTATGGATGATGAGATTCCATTGATACAGAGCCAATTCCAATAGACTTATTGAACGTTCCCATTGGCGTGCATCCAGCAGGAATTGAACCTACGAATTTGCCAATTATGAGTTGGGCGCTTTAACCATTCAGCCATGGATGCTTAACAGGGATCATCGTACATCGTGAATAACCAAATTCCAATTGAAATGAAATCTTTAGGAGGAATCAATGAAACGACATCAATTCAAATCCTGGATATTCGAATTGAGAGAGATATTGAGAGAGATCAAGAATTCTCACTATTTCTTAGATTCATGGATCAAATTCGATTCAGTGGGATCTTTCACTCACATTTT

>*Trillium smallii*

GACATTAGAAGTATATTGATTGTTCCCCAATAACCGAATACTTTTTTCTGTAAATACTGCATATTTGATTCCATCCATAAATCCATTTTCTTCCCTATGAGTTCCAGTATCGATAAGAATTCTAGTTCTTACTGTTCATATGTTATAGTATGAATATACCATATCAATTCGTTATGTATGGATGATGAGATTCCATTGATACAGAGCCAATTCCAATAGACTTATTGAACTGAACGTTCCATTGGCGTGCATCCAGCAGGAATTGAACCTACGAATTTGCCAATTATGAGTTGGGCGCTTTAACCATTCAGCCATGGATGCTTAACAGGTATTAT**^*^CATACTGAAACAGGTATTAT^*^CATACTGAAACAGGTATTAT^*^CATACTGAAACAGGTATTAT^*^CATACTGAAACAGGTATTAT^*^CATACTGAAACAGGTATTAT^*^CATACTGAAACAGGTATTAT^*^CATACTGAAACAGGTATTAT**CATAAATAACCAAATTCCAATTTAAATAAAGGAGGAAGTCATAGTCATGAAACGACATCAATTCAAATCCTGGATCTTCGAATTGAGAGAGATATTGAGAGAGATCAAGAATTCTCACTATTTCTTAGATTCATGGATCAAATTCGATTCAGTGGGATCTTTCACTCACATTTT

>*Trillium tschonoskii*

TTCCCCAATAACCGAATACTTTTTTCTGTAAATACTGCATATTTGATTCCATCCATAAATCCATTTTCTTCCCTATGAGTTCCAGTATCGATAAGAATTCTAGTTCTTACTGTTCATATGTTATAGTATGAATATACCATATCAATTCGTTATGTATGGATGATGAGATTCCATTGATACAGAGCCAATTCCAATAGACTTATTGAACTGAACGTTCCATTGGCGTGCATCCAGCAGGAATTGAACCTACGAATTTGCCAATTATGAGTTGGGCGCTTTAACCATTCAGCCATGGATGCTTAACAGGTATTAT**^*^CATACTGAAACAGGTATTAT^*^CATACTGAAACAGGTATTAT^*^CATACTGAAACAGGTATTAT^*^CATACTGAAACAGGTATTAT^*^CATACTGAAACAGGTATTAT^*^CATACTGAAACAGGTATTAT^*^CATACTGAAACAGGTATTAT**CATAAATAACCAAATTCCAATTTAAATAAAGGAGGAAGTCATAGTCATGAAACGACATCAATTCAAATCCTGGATCTTCGAATTGAGAGAGATATTGAGAGAGATCAAGAATTCTCACTATTTCTTAGATTCATGGATCAAATTCGATTCAGTGGGATCTTTCACTCACATTTT

>*Trillium camschatcense*

GACATTAGAAGTATATTGATTGTTCCCGAATAACCGAATACTTTTTTCTGTAAATACTGCATATTTGATTCCATCCATAAATCCATTTTCTTCCCTATGAGTTCCAGTATCGATAAGAATTCTAGTTCTTACTGTTCATATGTTATAGTATGAATATACCATATCAATTCGTTATGTATGGATGATGAGATTCCATTGATACAGAGCCAATTCCAATAGACTTATTGAACTGAACGTTCCATTGGCGTGCATCCAGCAGGAATTGAACCTACGAATTTGCCAATTATGAGTTGGGCGCTTTAACCATTCAGCCATGGATGCTTAACAGGTATTAT**^*^CATACTGAAACAGGTATTAT^*^CATACTGAAACAGGTATTAT^*^CATACTGAAACAGGTATTAT^*^CATACTGAAACAGGTATTAT^*^CATACTGAAACAGGTATTAT^*^CATACTGAAACAGGTATTAT^*^CATACTGAAACAGGTATTAT**CATAAATAACCAAATTCCAATTTAAATAAAGGAGGAAGTCATAGTCATGAAACGACATCAATTCAAATCCTGGATCTTCGAATTGAGAGAGATATTGAGAGAGATCAAGAATTCTCACTATTTCTTAGATTCATGGATCAAATTCGATTCAGTGGGATCTTTCACTCACATTTT

>*Trillium flexipes*

GACATTAGAAGTATATTGATTGTTCCCCAATAACCGAATACTTTTTTCTGTAAATACTGCATATTTGATTCCATCCATAAATCCATTTTCTTCCCTATGAGTTCCAGTATCGATAAGAATTCTAGTTCTTACTGTTCATATGTTATAGTATGAATATACCATATCAATTCGTTATGTATGGATGATGAGATTCCATTGATACAGAGCCAATTCCAATAGACTTATTGAACTGAACGTTCCATTGGCGTGCATCCAGCAGGAATTGAACCTACGAATTTGCCAATTATGAGTTGGGCGCTTTAACCATTCAGCCATGGATGCTTAACAGGTATTAT**^*^CATACTGAAACAGGTATTAT**CATACTGAAACATATTAT**^*^CATACTGAAACAGGTATTAT^*^CATACTGAAACAGGTATTAT^*^CATACTGAAACAGGTATTAT**CATACTGAAACATATTAT**^*^CATACTGAAACAGGTATTAT^*^CATACTGAAACAGGTATTAT^*^CATACTGAAACAGGTATTAT**CATACTGAAACATATTAT**^*^CATACTGAAACAGGTATTAT**CATACTGAAACATATTAT**^*^CATACTGAAACAGGTATTAT**CATACTGAAACATATTAT**^*^CATACTGAAACAGGTATTAT^*^CATACTGAAACAGGTATTAT^*^CATACTGAAACAGGTATTAT**CATACTGAAACATATTCCAATTTAAATAAAGGAGGAAGTAATAGTAATGAAACGACATCAATTCAAATCCTGGATCTTCGAATTGAGAGAGATATTGAGAGAGATCAAGAATTCTCACTATTTCTTAGATTCATGGATCAAATTCGATTCAGTGGGATCTTTCACTCACATTTT

>*Trillium rugelii*

GACATTAGAAGTATATTGATTGTTCCCCAATAACCGAATACTTTTTTCTGTAAATACTGCATATTTTATTCCATCCATAAATCCATTTTCTTCCCTATGAGTTCCAGTATCGATAAGAATTCTAGTTCTTACTGTTCATATGTTATAGTATGAATATACCATATCAATTCGTTATGTATGGATGATGAGATTCCATTGATACAGAGCCAATTCCAATAGACTTATTGAACTGAACGTTCCATTGGCGTGCATCCAGCAGGAATTGAACCTACGAATTTGCCAATTATGAGTTGGGCGCTTTAACCATTCAGCCATGGATGCTTAACAGGTATTAT**^*^CATACTGAAACAGGTATTAT**CATACTGAAACATATTAT**^*^CATACTGAAACAGGTATTAT**CATACTGAAACATATTATCATACTGAAACATATTAT**^*^CATACTGAAACAGGTATTAT**CATACTGAAACATATTAT**^*^CATACTGAAACAGGTATTAT**CATACTGAAACATATTAT**^*^CATACTGAAACAGGTATTAT**CATACTGAAACATATTATCATACTGAAACATATTAT**^*^CATACTGAAACAGGTATTAT**CATACTGAAACATATTAT**^*^CATACTGAAACAGGTATTAT**CATACTGAAACATATTAT**^*^CATACTGAAACAGGTATTAT^*^CATACTGAAACAGGTATTAT^*^CATACTGAAACAGGTATTAT**CATACTGAAACATATTCCAATTTAAATAAAGGAGGAAATAATAGTAATGAAACGACATCAATTCAAATCCTGGATCTTCGAATTGAGAGAGATATTGAGAGAGATCAAGAATTCTCACTATTTCTTAGATTCATGGATCAAATTCGATTCAGTGGGATCTTTCACTCACATTTT

>*Trillium erectum*

GACATTAGAAGTATATTGATTGTTCCCCAATAACCGAATACTTTTTTCTGTAAATACTGCATATTTGATTCCATCCATAAATCCATTTTCTTCCCTATGAGTTCCAGTATCGATAAGAATTCTAGTTCTTACTGTTCATATGTTATAGTATGAATATACCATATCAATTCGTTATGTATGGATGATGAGATTCCATTGATACAGAGCCAATTCCAATAGACTTATTGAACTGAACGTTCCATTGGCGTGCATCCAGCAGGAATTGAACCTACGAATTTGCCAATTATGAGTTGGGTGCTTTAACCATTCAGCCATGGATGCTTAACAGGTATTAT**^*^CATACTGAAACAGGTATTAT**CATACTGAAACATATTAT**^*^CATACTGAAACAGGTATTAT**CATACTGAAACATATTATCATACTGAAACATATTAT**^*^CATACTGAAACAGGTATTAT**CATACTGAAACATATTAT**^*^CATACTGAAACAGGTATTAT**CATACTGAAACATATTAT**^*^CATACTGAAACAGGTATTAT**CATACTGAAACATATTATCATACTGAAACATATTAT**^*^CATACTGAAACAGGTATTAT**CATACTGAAACATATTAT**^*^CATACTGAAACAGGTATTAT**CATACTGAAACATATTAT**^*^CATACTGAAACAGGTATTAT^*^CATACTGAAACAGGTATTAT^*^CATACTGAAACAGGTATTAT**CATACTGAAACATATTCCAATTTAAATAAAGGAGGAAATAATAGTAATGAAACGACATCAATTCAAATCCTGGATCTTCGAATTGAGAGAGATATTGAGAGAGATCAAGAATTCTCACTATTTCTTAGATTCATGGATCAAATTCGATTCAGTGGGATCTTTCACTCACATTTT

>*Trillium sulcatum*

GACATTAGAAGTATATTGATTGTTCCCCAATAACCGAATACTTTTTTCTGTAAATACTGCATATTTGATTCCATCCATAAATCCATTTTCTTCCCTATGAGTTCCAGTATCGATAAGAATTCTAGTTCTTACTGTTCATATGTTATAGTATGAATATACCATATCAATTCGTTATGTATGGATGATGAGATTCCATTGATACAGAGCCAATTCCAATAGACTTATTGAACTGAACGTTCCATTGGCGTGCATCCAGCAGGAATTGAACCTACGAATTTGCCAATTATGAGTTGGGTGCTTTAACCATTCAGCCATGGATGCTTAACAGGTATTAT**^*^CATACTGAAACAGGTATTAT**CATACTGAAACATATTAT**^*^CATACTGAAACAGGTATTAT**CATACTGAAACATATTATCATACTGAAACATATTAT**^*^CATACTGAAACAGGTATTAT**CATACTGAAACATATTAT**^*^CATACTGAAACAGGTATTAT**CATACTGAAACATATTAT**^*^CATACTGAAACAGGTATTAT**CATACTGAAACATATTATCATACTGAAACATATTAT**^*^CATACTGAAACAGGTATTAT**CATACTGAAACATATTAT**^*^CATACTGAAACAGGTATTAT**CATACTGAAACATATTAT**^*^CATACTGAAACAGGTATTAT^*^CATACTGAAACAGGTATTAT^*^CATACTGAAACAGGTATTAT**CATACTGAAACATATTCCAATTTAAATAAAGGAGGAAATAATAGTAATGAAACGACATCAATTCAAATCCTGGATCTTCGAATTGAGAGAGATATTGAGAGAGATCAAGAATTCTCACTATTTCTTAGATTCATGGATCAAATTCGATTCAGTGGGATCTTTCACTCACATTTT

>*Trillium simile*

GACATTAGAAGTATATTGATTGTTCCCCAATAACCGAATACTTTTTTCTGTAAATACTGCATATTTGATTCCATCCATAAATCCATTTTCTTCCCTATGAGTTCCAGTATCGATAAGAATTCTAGTTCTTACTGTTCATATGTTATAGTATGAATATACCATATCAATTCGTTATGTATGGATGATGAGATTCCATTGATACAGAGCCAATTCCAATAGACTTATTGAACTGAACGTTCCATTGGCGTGCATCCAGCAGGAATTGAACCTACGAATTTGCCAATTATGAGTTGGGCGCTTTAACCATTCATCCATGGATGCTTAACAGGTATTAT**^*^CATACTGAAACAGGTATTAT**CATACTGAAACATATTAT**^*^CATACTGAAACAGGTATTAT**CATACTGAAACATATTATCATACTGAAACATATTATCATACTGAAACATATTAT**^*^CATACTGAAACAGGTATTAT**CATACTGAAACATATTAT**^*^CATACTGAAACAGGTATTAT**CATACTGAAACATATTAT**^*^CATACTGAAACAGGTATTAT**CATACTGAAACATATTAT**^*^CATACTGAAACAGGTATTAT^*^CATACTGAAACAGGTATTAT**CATACTGAAACATATTAT**^*^CATACTGAAACAGGTATTAT^*^CATACTGAAACAGGTATTAT^*^CATACTGAAACAGGTATTAT**CATACTGAAACATATTCCAATTTAAATAAAGGAGGAAGTAATAGTAATGAAACGACATCAATTCAAATCCTGGATCTTCGAATTGAGAGAGATATTGAGAGAGATCAAGAATTCTCACTATTTCTTAGATTCATGGATCAAATTCGATTCAGTGGGATCTTTCACTCACATTTT

>*Paris dunniana*

GACATTAGAAGTATATTGATTGTTCCCCAATAACCGAATACTTTTTTCTGTAAATACTGCATATTTGATTCCATCCATAAATCCATTTTCTTCCCTATGAGTTCCAGTATCGATAAGAATTATAGTTCTTACTGTTCATATGTTATGGTATGAATATACCATATCAATTCGTTATGTATGGATGATGAGATTCCATTGATACAGAGCCAATTCCAATAGACTTATTGAACATTGGCGTGCATCCAGCAGGAATTGAACCTACGAATTTGCCAATTATGAGTTGGGCGCTTTAACCATTCAGCCATGGATGCTTAACAGGAATCATCGTA**^*^TATAACTTAACAGGAATCATCGTA^*^TATAACTTAACAGGAATCATCGTA^*^TATAACTTAACAGGAATCATCGTA^*^TATAACTTAACAGGAATCATCGTA**TCGTATATAAATATAATATATATATATAACCATAACCAAATTCCAATTTAAATTAAATCTTTAGGAGGAGGCAATGAAACGACATCAATTCAAATCCTGGATCTTCGAATTGAGAGAGATATTGAGAGAGATCAAGAATTCTCACTATTTCTTAGATTCATGGATCAAATTCAATTCAGTGGGATCTTTCACTCACATTTT

>*Paris thibetica*

GACATTAGAAGTATATTGATTGTTCCCCAATAACCGAATACTTTTTTCTGTAAATACTGCATATTTGATTCCATCCATAAATCCATTTTCTTCCCTATGAGTTCCAGTATCGATAAGAATTCTAGTTCTTACTGTTCATATGTTATGGTATGAATATACCATATCAATTCGTTATGTATGGATGATGAGATTCCATTGATACAGAGCCAATTCCAATAGACTTATTGAACATTGGCGTGCATCCAGCAGGAATTGAACCTACGAATTTGCCAATTATGAGTTGGGCGCTTTAACCATTCAGCCATGGATGCTTAACAGGAATCATCGTA**^*^TATAACTTAACAGGAATCATCGTA^*^TATAACTTAACAGGAATCATCGTA^*^TATAACTTAACAGGAATCATCGTA^*^TATAACTTAACAGGAATCATCGTA^*^TATAACTTAACAGGAATCATCGTA^*^TATAACTTAACAGGAATCATCGTA^*^TATAACTTAACAGGAATCATCGTA**TTCGTATATAAATATAATATATAACCAACCAAATTCCAATTTAAATTAAATCTTTAGGAGGAGGCAATGAAACGACATCAATTCAAATCTTGGATCTTCGAATTGAGAGAGATATTTAGAGAGATCAAGAATTCTCACTATTTCTTAGATTCATGGATCAAATTCAATTCAGTGGGATCTTTCACTCACATTTT

>*Paris axialis*

GACATTAGAAGTATATTGATTGTTCCCCAATAACCGAATACTTTTTTCTGTAAATACTGCATATTTGATTCCATCCATAAATCCATTTTCTTCCCTATGAGTTCCAGTATCGATAAGAATTCTAGTTCTTACTGTTCATATGTTATGGTATGAATATACCATATCAATTCGTTATGTATGGATGATGAGATTCCATTGATACAGAGCCAATTCCAATAGACTTATTGAACATTGGCGTGCATCCAGCAGGAATTGAACCTACGAATTTGCCAATTATGAGTTGGGCGCTTTAACCATTCAGCCATGGATGCTTAACAGGAATCATCGTATCGTA**^*^TATAACTTAACAGGAATCATCGTA^*^TATAACTTAACAGGAATCATCGTA^*^TATAACTTAACAGGAATCATCGTA^*^TATAACTTAACAGGAATCATCGTA^*^TATAACTTAACAGGAATCATCGTA^*^TATAACTTAACAGGAATCATCGTA**TATAACTTAACAGGAWTCATCGTATCGTATATAAATATAATATATATATATATAACCAATAACCAAATTCCAATTTAAATTAAATCTTTAGGAGGAGGCAATGAAACGACATCAATTCAAATCCTGGATCTTAGAATTGAGAGAGATATTGAGAGAGATCAAGAATTCTCACTATTTCTTAGATTCATGGATCAAATTCAATTCAGTGGGATCTTTCACTCACATTTT

>*Paris vietnamensis*

GACATTAGAAGTATATTGATTGTTCCCCAATAACCGAATACTTTTTTCTGTAAATACTGCATATTTGATTCCATCCATAAATCCATTTTCTTCCCTATGAGTTCCAGTATCGATAAGAATTCTAGTTCTTACTGTTCATATGTTATGGTATGAATATACCATATCAATTCGTTATGTATGGATGATGAGATTCCATTGATACAGAGCCAATTCCAATAGACTTATTGAACATTGGCGTGCATCCAGCAGGAATTGAACCTACGAATTTGCCAATTATGAGTTGGGCGCTTTAACCATTCAGCCATGGATGCTTAACAGGAATCATCGTATCGTA**^*^TATAACTTAACAGGAATCATCGTA^*^TATAACTTAACAGGAATCATCGTA^*^TATAACTTAACAGGAATCATCGTA^*^TATAACTTAACAGGAATCATCGTA^*^TATAACTTAACAGGAATCATCGTA^*^TATAACTTAACAGGAATCATCGTA^*^TATAACTTAACAGGAATCATCGTA**TCGTATATAAATATAATATATATATATATAACCAATAACCAAATTCCAATTTAAATTAAATCTTTAGGAGGAGGCAATGAAACGACATCAATTCAAATCCTGGATCTTAGAATTGAGAGAGATATTGAGAGAGATCAAGAATTCTCACTATTTCTTAGATTCATGGATCAAATTCAATTCAGTGGGATCTTTCACTCACATTTT

>*Paris polyphylla* var. *polyphylla*

GACATTAGAAGTATATTGATTGTTCCCCAATAACCGAATACTTTTTTCTGTAAATACTGCATATTTGATTCCATCCATAAATCCATTTTCTTCCCTATGAGTTCCAGTATCGATAAGAATTATAGTTCTTACTGTTCATATGTTATGGTATGAATATACCATATCAATTCGTTATGTATGGATGATGAGATTCCATTGATACAGAGCCAATTCCAATAGACTTATTGAACATTGGCGTGCATCCAGCAGGAATTGAACCTACGAATTTGCCAATTATGAGTTGGGCGCTTTAACCATTCAGCCATGGATGCTTAACAGGAATCATCGTA**^*^TATAACTTAACAGGAATCATCGTA^*^TATAACTTAACAGGAATCATCGTA^*^TATAACTTAACAGGAATCATCGTA^*^TATAACTTAACAGGAATCATCGTA^*^TATAACTTAACAGGAATCATCGTA^*^TATAACTTAACAGGAATCATCGTA^*^TATAACTTAACAGGAATCATCGTA^*^TATAACTTAACAGGAATCATCGTA**TCGTATATAAATATAATATATATATATAACCATTAACCATAACCAAATTCCAATTTAAATTAAATCTTTAGGAGGAGGCAATGAAACGACATCAATTCAAATCCTGGATCTTCGAATTGAGAGAGATATTGAGAGAGATCAAGAATTCTCACTATTTCTTAGATTCATGGATCAAATTCAATTCAGTGGGATCTTTCACTCACATTTT

>*Paris luquanensis*

GACATTAGAAGTATATTGATTGTTCCCCAATAACCGAATACTTTTTTCTGTAAATACTGCATATTTGATTCCATCCATAAATCCATTTTCTTCCCTATGAGTTCCAGTATCGATAAGAATTATAGTTCTTACTGTTCATATGTTATGGTATGAATATACCATATCAATTCGTTATGTATGGATGATGAGATTCCATTGATACAGAGCCAATTCCAATAGACTTATTGAACATTGGCGTGCATCCAGCAGGAATTGAACCTACGAATTTGCCAATTATGAGTTGGGCGCTTTAACCATTCAGCCATGGATGCTTAACAGGAATCATCGTA**^*^TATAACTTAACAGGAATCATCGTA^*^TATAACTTAACAGGAATCATCGTA^*^TATAACTTAACAGGAATCATCGTA^*^TATAACTTAACAGGAATCATCGTA^*^TATAACTTAACAGGAATCATCGTA^*^TATAACTTAACAGGAATCATCGTA^*^TATAACTTAACAGGAATCATCGTA^*^TATAACTTAACAGGAATCATCGTA^*^TATAACTTAACAGGAATCATCGTA^*^TATAACTTAACAGGAATCATCGTA^*^TATAACTTAACAGGAATCATCGTA^*^TATAACTTAACAGGAATCATCGTA^*^TATAACTTAACAGGAATCATCGTA**TCGTATATAAATATAATATATATATATAACCATAACCAAATTCCAATTTAAATTAAATCTTTAGGAGGAGGCAATGAAACGACATCAATTCAAATCCTGGATCTTCGAATTGAGAGAGATATTGAGAGAGATCAAGAATTCTCACTATTTCTTAGATTCATGGATCAAATTCAATTCAGTGGGATCTTTCACTCACATTTT

>*Paris polyphylla* var. *stenophylla*

GACATTAGAAGTATATTGATTGTTCCCCAATAACCGAATACTTTTTTCTGTAAATACTGCATATTTGATTCCATCCATAAATCCATTTTCTTCCCTATGAGTTCCAGTATCGATAAGAATTATAGTTCTTACTGTTCATATGTTATGGTATGAATATACCATATCAATTCGTTATGTATGGATGATGAGATTCCATTGATACAGAGCCAATTCCAATAGACTTATTGAACATTGGCGTGCATCCAGCAGGAATTGAACCTACGAATTTGCCAATTATGAGTTGGGCGCTTTAACCATTCAGCCATGGATGCTTAACAGGAATCATCGTA**^*^TATAACTTAACAGGAATCATCGTA^*^TATAACTTAACAGGAATCATCGTA^*^TATAACTTAACAGGAATCATCGTA^*^TATAACTTAACAGGAATCATCGTA^*^TATAACTTAACAGGAATCATCGTA^*^TATAACTTAACAGGAATCATCGTA^*^TATAACTTAACAGGAATCATCGTA^*^TATAACTTAACAGGAATCATCGTA^*^TATAACTTAACAGGAATCATCGTA^*^TATAACTTAACAGGAATCATCGTA^*^TATAACTTAACAGGAATCATCGTA^*^TATAACTTAACAGGAATCATCGTA^*^TATAACTTAACAGGAATCATCGTA**TCGTATATAAATATAATATATATATATAACCATAACCAAATTCCAATTTAAATTAAATCTTTAGGAGGAGGCAATGAAACGACATCAATTCAAATCCTGGATCTTCGAATTGAGAGAGATATTGAGAGAGATCAAGAATTCTCACTATTTCTTAGATTCATGGATCAAATTCAATTCAGTGGGATCTTTCACTCACATTTT

>*Paris polyphylla* var. *chinensis*

GACATTAGAAGTATATTGATTGTTCCCCAATAACCGAATACTTTTTTCTGTAAATACTGCATATTTGATTCCATCCATAAATCCATTTTCTTCCCTATGAGTTCCAGTATCGATAAGAATTATAGTTCTTACTGTTCATATGTTATGGTATGAATATACCATATCAATTCGTTATGTATGGATGATGAGATTCCATTGATACAGAGCCAATTCCAATAGACTTATTGAACATTGGCGTGCATCCAGCAGGAATTGAACCTACGAATTTGCCAATTATGAGTTGGGCGCTTTAACCATTCAGCCATGGATGCTTAACAGGAATCATCGTA**^*^TATAACTTAACAGGAATCATCGTA^*^TATAACTTAACAGGAATCATCGTA^*^TATAACTTAACAGGAATCATCGTA^*^TATAACTTAACAGGAATCATCGTA^*^TATAACTTAACAGGAATCATCGTA^*^TATAACTTAACAGGAATCATCGTA^*^TATAACTTAACAGGAATCATCGTA^*^TATAACTTAACAGGAATCATCGTA^*^TATAACTTAACAGGAATCATCGTA^*^TATAACTTAACAGGAATCATCGTA^*^TATAACTTAACAGGAATCATCGTA^*^TATAACTTAACAGGAATCATCGTA**TCGTATATAAATATAATATATATATATAACCATAACCAAATTCCAATTTAAATTAAATCTTTAGGAGGAGGCAATGAAACGACATCAATTCAAATCCTGGATCTTCGAATTGAGAGAGATATTGAGAGAGATCAAGAATTCTCACTATTTCTTAGATTCATGGATCAAATTCAATTCAGTGGGATCTTTCACTCACATTTT

>*Paris mairei*

GACATTAGAAGTATATTGATTGTTCCCCAATAACCGAATACTTTTTTCTGTAAATACTGCATATTTGATTCCATCCATAAATCCATTTTCTTCCCTATGAGTTCCAGTATCGATAAGAATTATAGTTCTTACTGTTCATATGTTATGGTATGAATATACCATATCAATTCGTTATGTATGGATGATGAGATTCCATTGATACAGAGCCAATTCCAATAGACTTATTGAACATTGGCGTGCATCCAGCAGGAATTGAACCTACGAATTTGCCAATTATGAGTTGGGCGCTTTAACCATTCAGCCATGGATGCTTAACAGGAATCATCGTA**^*^TATAACTTAACAGGAATCATCGTA^*^TATAACTTAACAGGAATCATCGTA^*^TATAACTTAACAGGAATCATCGTA^*^TATAACTTAACAGGAATCATCGTA^*^TATAACTTAACAGGAATCATCGTA^*^TATAACTTAACAGGAATCATCGTA^*^TATAACTTAACAGGAATCATCGTA^*^TATAACTTAACAGGAATCATCGTA^*^TATAACTTAACAGGAATCATCGTA^*^TATAACTTAACAGGAATCATCGTA^*^TATAACTTAACAGGAATCATCGTA^*^TATAACTTAACAGGAATCATCGTA**TCGTATATAAATATAATATATATATATAACCATAACCAAATTCCAATTTAAATTAAATCTTTAGGAGGAGGCAATGAAACGACATCAATTCAAATCCTGGATCTTCGAATTGAGAGAGATATTGAGAGAGATCAAGAATTCTCACTATTTCTTAGATTCATGGATCAAATTCAATTCAGTGGGATCTTTCACTCACATTTT

>*Paris rugosa*

GACATTAGAAGTATATTGATTGTTCCCCAATAACCGAATACTTTTTTCTGTAAATACTGCATATTTGATTCCATCCATAAATCCATTTTCTTCCCTATGAGTTCCAGTATCGATAAGAATTCTAGTTCTTACTGTTCATATGTTATGGTATGAATATACCATATCAATTCGTTATGTATGGATGATGAGATTCCATTGATACAGAGCCAATTCCAATAGACTTATTGAACATTGGCGTGCATCCAGCAGGAATTGAACCTACGAATTTGCCAATTATGAGTTGGGCGCTTTAACCATTCAGCCATGGATGCTTAACAGGAATCATCGTA**^*^TATAACTTAACAGGAATCATCGTA^*^TATAACTTAACAGGAATCATCGTA^*^TATAACTTAACAGGAATCATCGTA^*^TATAACTTAACAGGAATCATCGTA^*^TATAACTTAACAGGAATCATCGTA^*^TATAACTTAACAGGAATCATCGTA^*^TATAACTTAACAGGAATCATCGTA^*^TATAACTTAACAGGAATCATCGTA^*^TATAACTTAACAGGAATCATCGTA^*^TATAACTTAACAGGAATCATCGTA^*^TATAACTTAACAGGAATCATCGTA^*^TATAACTTAACAGGAATCATCGTA**TCGTATATAAATATAATATATATATATAACCATAACCAAATTCCAATTTAAATTAAATCTTTAGGAGGAGGCAATGAAACGACATCAATTCAAATCCTTGATCTTCGAATTGAGAGAGATATTGAGAGAGATCCAGAATTCTCACTATTTCTTAGATTCATGGATCAAATTCAATTCAGTGGGATCTTTCACTCACATTTT

>*Paris fargesii*

GACATTAGAAGTATATTGATTGTTCCCCAATAACCGAATACTTTTTTCTGTAAATACTGCATATTTGATTCCATCCATAAATCCATTTTCTTCCCTATGAGTTCCAGTATCGATAAGAATTATAGTTCTTACTGTTCATATGTTATGGTATGAATATACCATATCAATTCGTTATGTATGGATGATGAGATTCCATTGATACAGAGCCAATTCCAATAGACTTATTGAACATTGGCGTGCATCCAGCAGGAATTGAACCTACGAATTTGCCAATTATGAGTTGGGCGCTTTAACCATTCAGCCATGGATGCTTAACAGGAATCATCGTA**^*^TATAACTTAACAGGAATCATCGTA^*^TATAACTTAACAGGAATCATCGTA^*^TATAACTTAACAGGAATCATCGTA^*^TATAACTTAACAGGAATCATCGTA^*^TATAACTTAACAGGAATCATCGTA^*^TATAACTTAACAGGAATCATCGTA^*^TATAACTTAACAGGAATCATCGTA^*^TATAACTTAACAGGAATCATCGTA^*^TATAACTTAACAGGAATCATCGTA^*^TATAACTTAACAGGAATCATCGTA^*^TATAACTTAACAGGAATCATCGTA^*^TATAACTTAACAGGAATCATCGTA^*^TATAACTTAACAGGAATCATCGTA^*^TATAACTTAACAGGAATCATCGTA^*^TATAACTTAACAGGAATCATCGTA^*^TATAACTTAACAGGAATCATCGTA**TCGTATATAAATATAATATATATATATAACCATAACCAAATTCCAATTTAAATTAAATCTTTAGGAGGAGGCAATGAAACGACATCAATTCAAATCCTGGATCTTCGAATTGAGAGAGATATTGAGAGAGATCAAGAATTCTCACTATTTCTTAGATTCATGGATCAAATTCAATTCAGTGGGATCTTTCACTCACATTTT

**Type B**

>*Trillium maculatum*

GTTGACCCTGATTCGACATTAGAAGTATATTGATTGTTCCCCAATAACCGAATACTTTTTTCTGTAAATACTGCATATTTGATTCCATCCATAAATCCATTTTCTTCCCTATGAGTTCCAGTATCGATAAGAATTCTAGTTCTTACTGTTCATATGTTATGGTACGAATATACTATACCCTATCAATTCGTTATGTATGGATGATGAGATTCCATTGATACAGAGC**^*^CAATTCCAATAGACTTGACTTATGAAACAGTTTCCAATAGAATTGAGAATTGACTTATGAGACGGTTCCATTGGTGTGCATCCAGTAGGAATCGAACCTACGGATTTGCCAATTATGAGTTGGGCGCTTTAACCATTCAGCCATGGATGCTTAACAGGGATTCTCAATTATCATCAATAA**CCAAATT**^*^CAATTCCAATAGACTTGACTTATGAAACAGTTTCCAATAGAATTGAGAATTGACTTATGAGACGGTTCCATTGGTGTGCATCCAGTAGGAATCGAACCTACGGATTTGCCAATTATGAGTTGGGCGCTTTAACCATTCAGCCATGGATGCTTAACAGGGATTCTCAATTATCATCAATAA**TATCATAACATAAATAACCCAATTCCAATTTTCTTCATCAAATCTTTAGGAGGAAGTAATAGTAATGCAACGACATCAATTCAAATCCTGGATCTTCGAATTGAGAGAGATATTGAGAGAGATCAAGAATTCTCACTATTTCTTAGATTCATGGATCAAATTCGATTCAGTGGGATCTTTCACTCACATTTTTTTTCACCAAGAACGCTTTATGAAACTCTTTGACCCC

>*Trillium sessile*

GACATTAGAAGTATATTGATTGTTCCCCAATAACCGAATACTTTTTTCTGTAAATACTGCATATTTGATTCCATCCATAAATCCATTTTCTTCCCTATGAGTTCCAGTATCGATAAGAATTCTAGTTCTTACTGTTCATATGTTATGGTACGAATATACTATACCCTATCAATTCGTTATGTATGGATGATGAGATTCCATTGATACAGAGC**^*^CAATTCCAATAGACTTGACTTATGAAACAGTTTCCAATAGAATTGAGAATTGACTTATGAGACGGTTCCATTGGTGTGCATCCAGTAGGAATCGAACCTACGGATTTGCCAATTATGAGTTGGGCGCTTTAACCATTCAGCCATGGATGCTTAACAGGGATTCTCAATTATCATCAATAA**CCAAATT**^*^CAATTCCAATAGACTTGACTTATGAAACAGTTTCCAATAGAATTGAGAATTGACTTATGAGACGGTTCCATTGGTGTGCATCCAGTAGGAATCGAACCTACGGATTTGCCAATTATGAGTTGGGCGCTTTAACCATTCAGCCATGGATGCTTAACAGGGATTCTCAATTATCATCAATAA**TATCATAACATAAATAACCCAATTCCAATTTTCTTCATCAAATCTTTAGGAGGAAGTAATAGTAATGCAACGACATCAATTCAAATCCTGGATCTTCGAATTGAGAGAGATATTGAGAGAGATCAAGAATTCTCACTATTTCTTAGATTCATGGATCAAATTCGATTCAGTGGGATCTTTCACTCACATTTT

>*Trillium luteum*

GACATTAGAAGTATATTGATTGTTCCCCAATAACCGAATACTTTTTTCTGTAAATACTGCATATTTGATTCCATCCATAAATCCATTTTCTTCCCTATGAGTTCCAGTATCGATAAGAATTCTAGTTCTTACTGCTCATATGTTATGGTACGAATATACTATACCCTATCAATTCGTTATGTATGGATGATGAGATTCCATTGATACAGAGC**^*^CAATTCCAATAGACTTGACTTATGAAACAGTTTCCAATAGAATTGAGAATTGACTTATGAGACGGTTCCATTGGTGTGCATCCAGTAGGAATCGAACCTACGGATTTGCCAATTATGAGTTGGGCGCTTTAACCATTCAGCCATGGATGCTTAACAGGGATTCTCAATTATCATCAATAA**CCAAATT**^*^CAATTCCAATAGACTTGACTTATGAAACAGTTTCCAATAGAATTGAGAATTGACTTATGAGACGGTTCCATTGGTGTGCATCCAGTAGGAATCGAACCTACGGATTTGCCAATTATGAGTTGGGCGCTTTAACCATTCAGCCATGGATGCTTAACAGGGATTCTCAATTATCATCAATAA**TATCATAACATAAATAACCCAATTCCAATTTTCTTCATCAAATCTTTAGGAGGAAGTAATAGTAATGCAACGACATCAATTCAAATCCTGGATCTTCGAATTGAGAGAGATATTGAGAGAGATCAAGAATTCTCACTATTTCTTAGATTCATGGATCAAATTCGATTCAGTGGGATCTTTCACTCACATTTT

>*Trillium chloropetalum*

TAACCGAATACTTTTTTCTGTAAATACTGCATATTTGATTCCATCCATAAATCCATTTTCTTCCCTATGAGTTCCAGTATCGATAAGAATTCTAGTTCTTACTGTTCATATGTTATGGTACGAATATACCCTATCAATTCGTTATGTATGGATGATGAGATTCCATTGATACAGAGC**^*^CAATTCCAATAGACTTGACTTATGAAACAGTTTCCAATAGAATTGAGAATTGACTTATGAGACGGTTCCATTGGTGTGCATCCAGTAGGAATCGAACCTACGGATTTGCCAATTATGAGTTGGGCGCTTTAACCATTCAGCCATGGATGCTTAACAGGGATTCTCAATTATCCATCAATAATATCATAACATAAATAACC^*^CAATTCCAATAGACTTGACTTATGAAACAGTTTCCAATAGAATTGAGAATTGACTTATGAGACGGTTCCATTGGTGTGCATCCAGTAGGAATCGAACCTACGGATTTGCCAATTATGAGTTGGGCGCTTTAACCATTCAGCCATGGATGCTTAACAGGGATTCTCAATTATCATCAATAATATCATAACATAAATAACC**CAATTCCAATTTTCTTCATCAAATCTTTAAATCTTTAGGAGGAAGTAATAGTAATGCAACGACATCAATTCAAATCCTGGATCTTCGAATTGAGAGAGATATTGAGAGAGATCAAGAATTCTCACTATTTCTTAGATTCATGGATCAAATTCGATTCAGTGGGATCTTTCACTCACATTTT

>*Trillium underwoodii*

GATTGTTCCCCAATAACCGAATACTTTTTTCTGTAAATACTGCATATTTGATTCCATCCATAAATCCATTTTCTTCCCTATGAGTTCCAGTATCGATAAGAATTCTAGTTCTTACTGTTCATATGTTATGGTATGAATATACCCTATCAATTCGTTATGTATGGATGATGAGATTCCATTGATACAGAGC**^*^CAATTCCAATAGACTTGACTTATGAAACAGTTTCCAATAGAATTGAGAATTGACTTATGAGACGGTTCCATTGGTGTGCATCCAGTAGGAATCGAACCTACGGATTTGCCAATTAAGAGTTGGGCGCTTTAACCATTCAGCCATGGATGCTTAACAGGGATTCTCAATTATCCTAAATAATATCATAACTATAACATAAATAACC**AAATT**^*^CAATTCCAATAGACTTGACTTATGAAACAGTTTCCAATAGAATTGAGAATTGACTTATGAGACGGTTCCATTGGTGTGCATCCAGTAGGAATCGAACCTACGGATTTGCCAATTAAGAGTTGGGCGCTTTAACCATTCAGCCATGGATGCTTAA**CTTAACCTTAA**CAGGGATTCTCAATTATCCTAAATAATATCATAATATAACTATAACATAAATAACC**CAATTCCAATTTTTATCAAATCTTTAGGAGGAAGTAATAGTAATGCAACGACATCAATTCAAATCCTGGATCTTCGAATTGAGAGAGATATTGAGAGAGATCAAGAATTCTCACTATTTCTTAGATTCATGGATCAAATTCTATTCAGTGGGATCTTTCACTCACATTTT

>*Paris japonica*

GACATTAGAAGTATATTGATTGTTCCCCAATAACCGAATACTTTTTTCTGTAAATACTGCATATTTGATTCCATCCATAAATCCATTTTCTTCCCTATGAGTTCCAGTATCGATAAGAATTCTAGTTCTTACTGTTCATATGTTATGGTATGAATATACCATATCAATTCGTTATGTATGGATGATGAGATTCCATTGATACAGAGCCAATTCCAATAGACTTATTGAACATTGGCGTGCATCCAGCAGGAATTGAACCTACGAATTTGCCAATTATGAGTTGGGCGCTTTAACCATTCAGCCATGGATGCTTAACACTTAACAGGAATCATCGTATA**^*^TAAATAGAATATAACTAACCAAATTCCAATTGACTTATTGAACACAGGAATCATCGTATATAAATAGAATATAACTAACCAAATTCGAATTGACTTATTGAACATTGGAATCATCGTAAATAGAATATAACTAACGAAATTCCAAT**TGACTTATTGAACATTGGAATCATCG**^*^TAAATAGAATATAACTAACCAAATTCCAATTGACTTATTGAACACAGGAATCATCGTATATAAATAGAATATAACTAACCAAATTCGAATTGACTTATTGAACATTGGAATCATCGTAAATAGAATATAACTAACGAAATTCCAAT**TGACTTATTGAACATTGGAATCATCG**^*^TAAATAGAATATAACTAACCAAATTCCAATTGACTTATTGAACACAGGAATCATCGTATATAAATAGAATATAACTAACCAAATTCGAATTGACTTATTGAACATTGGAATCATCGTAAATAGAATATAACTAACGAAATTCCAAT**AGACTTATTGAACATTGGCGTGCATCCAGCAGGAATCGAACCTACGAATTTGCCAATTATGAGTTGGGCGCTTTAACCATTCAGCCATGGATGCTTAACACTTAACAGGAATCATCGTATATAAATAGAATATAACTAACCAAATTTGAATTGACTTATTGAACATTGGAATCATCGTAAATAGAATATAACTAACGAAATTCCAATTTAAATTCAATCTTTAAGATTTAGGAGGAGACAATGAAACGACATCAATTCAAATACTGGATCTTCGAATTGAGAGAGATACTGAGAGAGATCAAGAATTCTCACTATTT

**Type C**

>*Trillium govanianum*

TGATTGTTCCCCAATAACCGAATACTTTTTTCTGTAAATACTGCATATTTGATTCCATCCATAAATCCATTTTCTTCCCTATGAGTTCCAGTATCGATAAGAATTCTAGTTCTTACTGTTCATATGTTATGGTATGAATATACCATATCAATTCGTTATGTATGGATGATGAGATTCC**^*^ATTGATACAGAGCCAATTCCAATAGACTTATTGAACGTTCCATTGGCGTGCATCCAGCAGGAATTGAACCTACGAATTTGCCAATTATGAGTTGGGCGCTTTAACCATTCAGCCATGGATGCTTAACAGGGATTATCGTAAATAACCAAATTCC^*^ATTGATACAGAGCCAATTCCAATAGACTTATTGAACGTTCCATTAGCGTGCATCCAGCAGGAATTGAACCTACGAATTTGCCAATTATGAGTTGGGCGCTTTAACCATTCAGCCATGGATGCTTAACAGGGATTATCGTAAATAACCAAATTCC**A**ATTGATACAGAGCCAATTCCAATAGACTTATTGAACGTTCC^*^ATTAGCGTGCATCCAGCAGGAATTGAACCTACGAATTTGCCAATTATGAGTTGGGCGCTTTAACCATTCAGCCATGGATGCTTAACAGGGATTATCGTAAATAACCAAATTCC**AATTGAAATAAAATATTTAGGAGGAAACAAACAATGAAACGACATCAATTCAAATCCTGGATCTTCGAATTGAGAGAGATATTGAGAGAGATCAAGAATTCTCACTATTTCTTAGATTCATGGATCAAATTCGATTCAGTGGGATCTTTCACTCACATTTT

>*Paris quadrifolia*

GACATTAGAAGTATATTGATTGTTTCCCAATAACCGAATACTTTTTTCTGTAAATACTGCATATTTGATTCCATCCATAAATCCATTTTCTTCCCTATGAGTTCCAGTATCGATAAGAATTCTAGTTCTTACTGTTCATATGTTATGGTATGAATATACCATATCAATTCGTTATGTATGGATGC**^*^TTAACAGGAATCATCGTAAATAAAATAACCAAATTCCAATAGACTTATTGAACGTTCCATTGGCGTGCATCCAGCAGGAATTGAACCTACGAATTTGCCAATTATGAGTTGGGCGCTTTAACCATTCAGCCATGGATGC^*^TTAACAGGAATCATCGTAAATAAAATAACCAAATTCCAATAGACTTATTGAACGTTCCATTGGCGTGCATCCAGCAGGAATTGAACCTACGAATTTGCCAATTATGAGTTGGGCGCTTTAACCATTCAGCCATGGATGC^*^TTAACAGGAATCATCGTAAATAAAATAACCAAATTCCAATAGACTTATTGAACGTTCCGTTGGCGTGCATCCAGCAGGAATTGAACCTACGAATTTGCCAATTATGAGTTGGGCGCTTTAACCATTCAGCCATGGATGC**TTAACAGGTATCATCATAAATAACCAAATTCCAATTTAAATGAAATCTTTAGGAGGAAGCAATAGCAATGAAACGACATCAATATCAATTCAAATCCTGGATCTTCGAATTGAGAGAAATAGTGAGAGAGATCAAGAATTCTCACTATTTCTTAGATTCATGGATCAAATTCGATTCAGTGGGATCTTTCACTCACATTTT

>*Paris verticillata*

GTTGATCCTGATTCGACATTAGAAGTATATTGATTGTTTCCCAATAACCGAATACTTTTTTCTGTAAATACTGCATATTTGATTCCATCCATAAATCCATTTTCTTCCCTATGAGTTCCAGTATCGATAAGAATTCTAGTTCTTACTGTTCATATGTTATGGTATGAATATACCATATCAATTCGTTATGT**^*^ATGGATGCTTAACAGGAATCATCGTAAATAAAATAACCAAATTCCAATAGACTTATTGAACGTTCCATTGGCGTGCATCCAGCAGGAATTGAACCTACGAATTTGCCAATTATGAGTTGGGCGCTTTAACCATTC**AGCC**^*^ATGGATGCTTAACAGGAATCATCGTAAATAAAATAACCAAATTCCAATAGACTTATTGAACGTTCCGTTGGCGTGCATCCAGCAGGAATTGAACCTACGAATTTGCCAATTATGAGTTGGGCGCTTTAACCATTC**AGCC**^*^ATGGATGCTTAACAGGAATCATCGTAAATAAAATAACCAAATTCCAATAGACTTATTGAACGTTCCGTTGGCGTGCATCCAGCAGGAATTGAACCTACGAATTTGCCAATTATGAGTTGGGCGCTTTAACCATTC**AGCCATGGATGCTTAACAGGTATCATCATAAATAACCAAATTCCAATTTAAATGAAATCTTTAGGAGGAAGCAATAGCAATGAAACGACATCAATATCAATTCAAATCCTGGATCTTCGAATTGAGAGAAATAGTGAGAGAGATCAAGAATTCTCACTATTTCTTAGATTCATGGATCAAATTCGATTCAGTGGGATCTTTCACTCACATTTTT

>*Paris incompleta*

GACATTAGAAGTATATTGATTGTTCCCCAATAACCGAATACTTTTTTCTGTAAATACTGCATATTTGATTCCATCCATAAATCCATTTTCTTCCCTATGAGTTCCAGTATCGATAAGAATTCTAGTTCTTACTGTTCATATGTTATAGTATGAATATACCATATCAATTCGTTATGTATGGATGATGAGATTCCATTGATACAGAG**CCAATTCAAATAGACTTATTGAACGTTCCACTGGCGTGCATCCAGCAGGAATTGAACCTACGAATTTGCCAATTATGAGTTGGGCGCTTTAACCATTCAGCCATGGATGCTTAACACTTTACAGGAATGTAAATATAATATATAATATAAATAA^*^CCAAATTCCAATAGACTTATTGAACGTTCCATTGGCGTGCATCCAGCAGGAATTGAACCTACGAATTTGCCAATTATGAGTTGGGCGCTTTAACCATTCAGCCATGGATGCTTAACACTTTACAGGAATGTAAATATAATATATAATATAAATAA^*^CCAAATTCCAATAGACTTATTGAACGTTCCATTGGCGTGCATCCAGCAGGAATTGAACCTACGAATTTGCCAATTATGAGTTGGGCGCTTTAACCATTCAGCCATGGATGCTTAACACTTAACAGGAATGTAAATATAATATATAATATATATAAATAA**CCAAATTCCAATTTTAATGAAATATTTAGGAGGAAACAATATCAATGAAAAGACATCAATATCAATTCAAATCCTGGATCTTCGAATTGAGAGAGATATTTAGAGAGATCAAGAATTCTCACTATTTCTTAGATTCATGGATCAAATTCAATTCAGTGGGATCTTTCACTCACATTTT
